# Supplementary material for: Synthesis of Carvone-Derived 1,2,3-Triazoles Study of Their Antioxidant Properties and Interaction with Bovine Serum Albumin
Source: Molecules. 2018 Nov 16;23(11):2991. doi: 10.3390/molecules23112991 (PMC6278498; doi:10.3390/molecules23112991)
Supplement: Supplementary file 1 [file molecules-23-02991-s001.pdf]

Supplementary Materials

Synthesis of Carvone derived 1,2,3-Triazoles. Study of their Antioxidant Properties and Interaction with Bovine Serum Albumin

Armen S. Galstyan [1]*, Armen I. Martiryan[1], Karine R. Grigoryan[1], Armine G. Ghazaryan[1], Melanya A. Samvelyan[1], Tariel V. Ghochikyan[1] and Valentine G. Nenajdenko[2]*

1. *Fluorescence Spectroscopy Studies*

BSA purchased from the Sigma Chemical. Co. USA was used without further purification. The concentration of BSA (0.4 mg/ml) was determined spectrophotometrically using a molar extinction coefficient 39080M^{-1} cm^{-1} at 280 nm. The concentration of **4e** was varied in the range of 4.00x10^{-5} - 2.00x10^{-4}M. The protein solutions were prepared in saline solution. The fluorescence spectra were recorded on a Cary Eclipse (Varian) spectrofluorometer at 298 and 308K, in the range λ = 300-500 nm at the excitation wavelength λ = 280 nm. The temperature of the samples was maintained constant by circulating the thermostated water connected to the spectrophotometer of the LAUDA Alpha 100 thermostat (Germany). l = 1 cm cuvettes were used. The graphs were constructed and analyzed using the ORIGIN 8.0 software.

*1.1. Determination of the thermodynamic binding parameters of BSA - **4e***

The character of intermolecular interactions between the biomolecule and the ligand was determined by the relationship of thermodynamic parameters (ΔH, ΔS and ΔG). ΔG shows the possibility of spontaneous rate of the reaction, and ΔH and ΔS are the main criteria for confirming the character of interactions. The thermodynamic parameters of binding with BSA are determined by the following equations:

$$\Delta G = -RT \ln K_b \quad (1),$$

$$\ln \frac{K_{b_1}}{K_{b_2}} = \left(\frac{1}{T_1} - \frac{1}{T_2}\right)\frac{\Delta H}{R} \quad (2),$$

$$\Delta G = \Delta H - T\Delta S \quad (3),$$

where K_{b_1} and K_{b_2} are the binding constants at temperatures of 298 and 303K, and R is the gas constant. The obtained results are presented in Table 4.

The binding constant and the number of binding sites (n) with BSA were determined based on the data of quenching of BSA fluorescence according to the well-known [Lakowicz, J. Principles of Fluorescence Spectroscopy, 3rd ed., New York: Springer, 2006, 529.] equation:

$$\lg[(F_0 - F)/F] = \lg K_b + n\lg[Q] \quad (4),$$

where F_0 and F are the fluorescence intensities of BSA in the absence and presence of **4e**, respectively; K_b is the binding constant, n – is the number if binding sites. *[Q]* is the concentration of quencher-**4e**. K_b and n are determined from the dependence $\lg(F_0 - F)/F$ vs lg[Q]. Figure 7 shows a plot of log $(F_0 - F)/F$ vs log [Q] at two different temperatures.

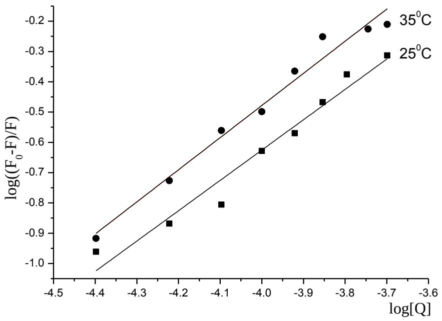

Figure 7. The plot of log ($F_0 - F$) / F versus log [Q] at two different temperatures.

1.2. Determination of the distance between the protein and ligand - ***4e***

The distance between the protein and the ligand can be calculated using the theory of resonance energy transfer (Foerster theory) [Lakowicz, J. Principles of Fluorescence Spectroscopy, 3rd ed., New York: Springer, 2006, 529.]. The efficiency of energy transfer E is calculated using the equation:

$$E = 1 - \frac{F}{F_0} = \frac{R_0^6}{R_0^6 + r^6} \quad (5),$$

where E is the energy transfer efficiency, F_0 and F are the fluorescence intensities of BSA in the absence and presence of **4e**, respectively; r is the distance between the donor and the acceptor, R_0 is the Foerster radius, when the energy transfer efficiency is 50% and can be calculated by the following equation:

$$R_0^6 = 8.79 \times 10^{-25} \kappa^2 n^{-4} \Phi J \quad (6),$$

where κ^2 - is the spatial orientation factor (κ^2 = 2/3); n – is the refractive index (1.336); Φ is the fluorescence quantum yield of the donor, J is the overlap integral of the donor fluorescence spectrum and the acceptor absorption spectrum.

$$J = \sum F(\lambda)\ \varepsilon(\lambda)\ \lambda^4 \Delta\lambda) / \sum F(\lambda)\Delta\lambda \quad (7)$$

where $F(\lambda)$ is the fluorescence intensity of the donor at wavelength λ, ε (λ) is the molar extinction coefficient of acceptor at wavelength λ. Using equations (5) - (7), the overlap integral, the energy transfer efficiency, the Foerster radius, and the distance between the donor and the acceptor were calculated and given in Table.

Table. The values of the overlap integral, the energy transfer efficiency, the Forster radius and the distance between the BSA and **4e**.

T, K	*I*, $cm^3 \cdot l \cdot mol^{-1}$	*E*	R_0, nm	*r, nm*
298	$1.79 x 10^{-14}$	0.019	3.778	7.27
308	$1.80 x 10^{-14}$	0.043	3.782	7.06

2. NMR Spectrum of Compounds **3a-i**, **10-N_3-Car**. and **4a-i**

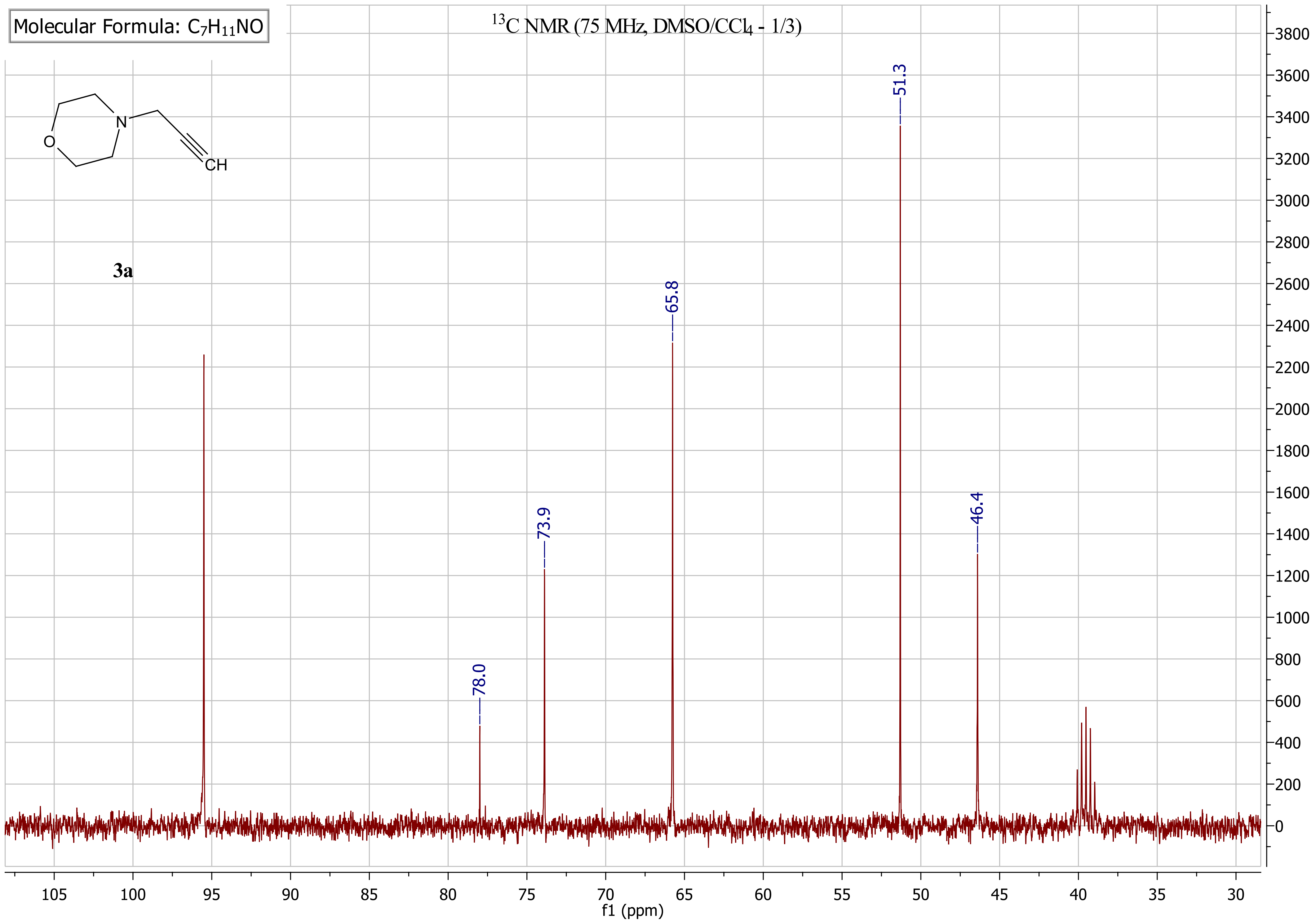

Molecular Formula: $C_7H_{11}NO$
^{13}C NMR (75 MHz, DMSO/CCl_4 - 1/3)
O
N
CH
3a
78.0
73.9
65.8
51.3
46.4
f1 (ppm)

Molecular Formula: $C_7H_{11}NO$

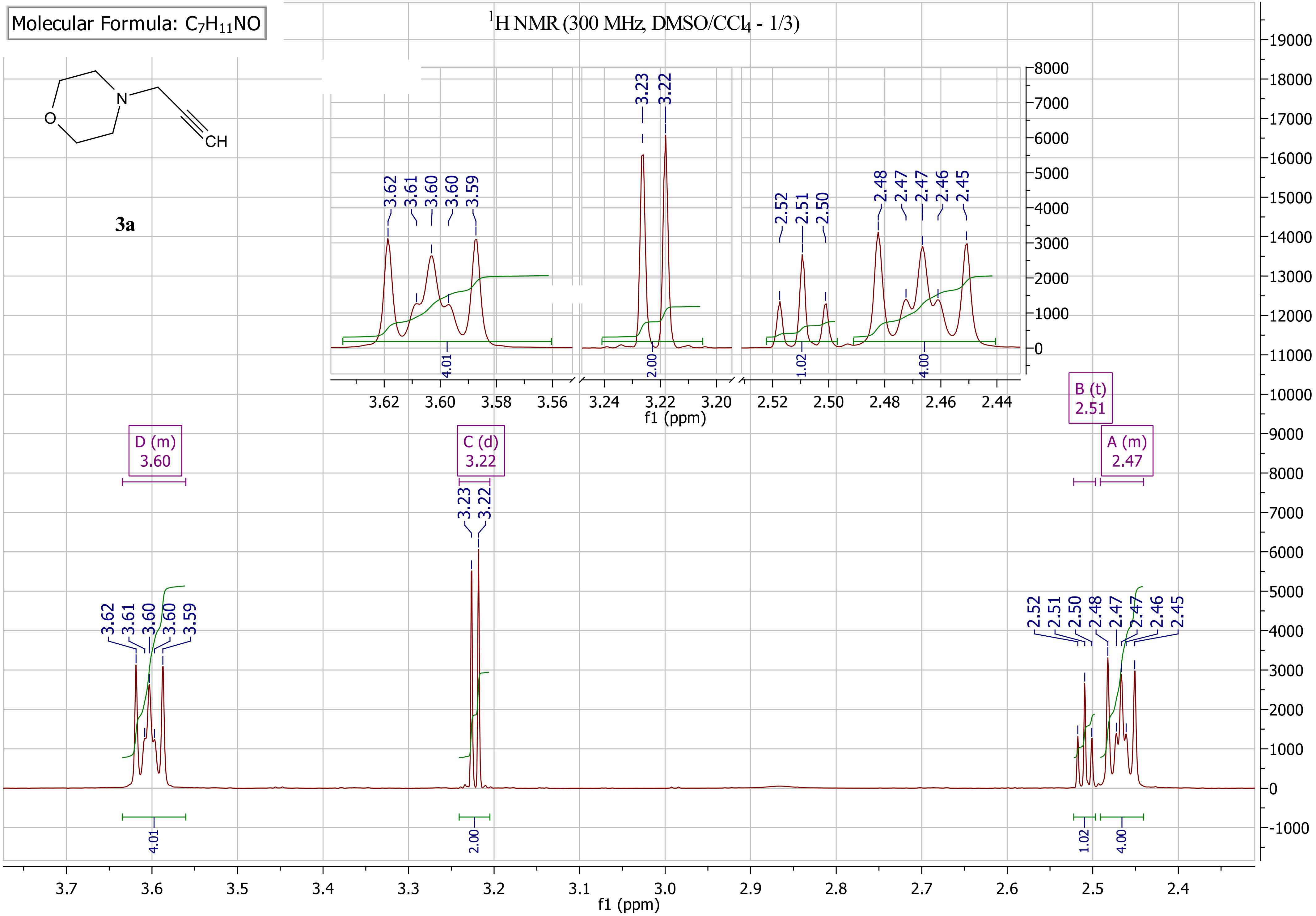

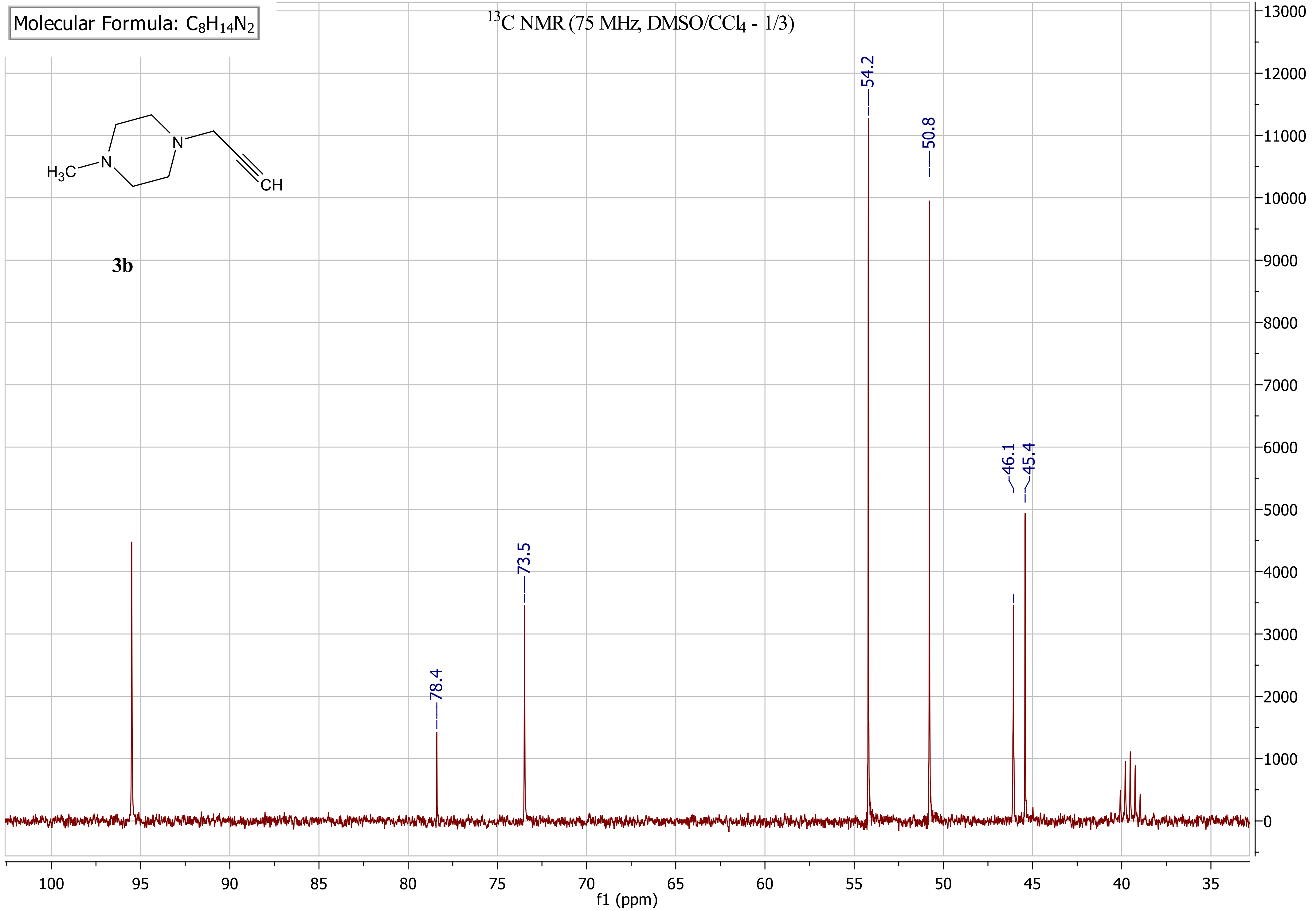
Molecular Formula: $C_8H_{14}N_2$
^{13}C NMR (75 MHz, DMSO/CCl_4 - 1/3)
H_3C
N
N
CH
3b
54.2
50.8
46.1
45.4
73.5
78.4
f1 (ppm)
100
95
90
85
80
75
70
65
60
55
50
45
40
35
13000
12000
11000
10000
9000
8000
7000
6000
5000
4000
3000
2000
1000
0

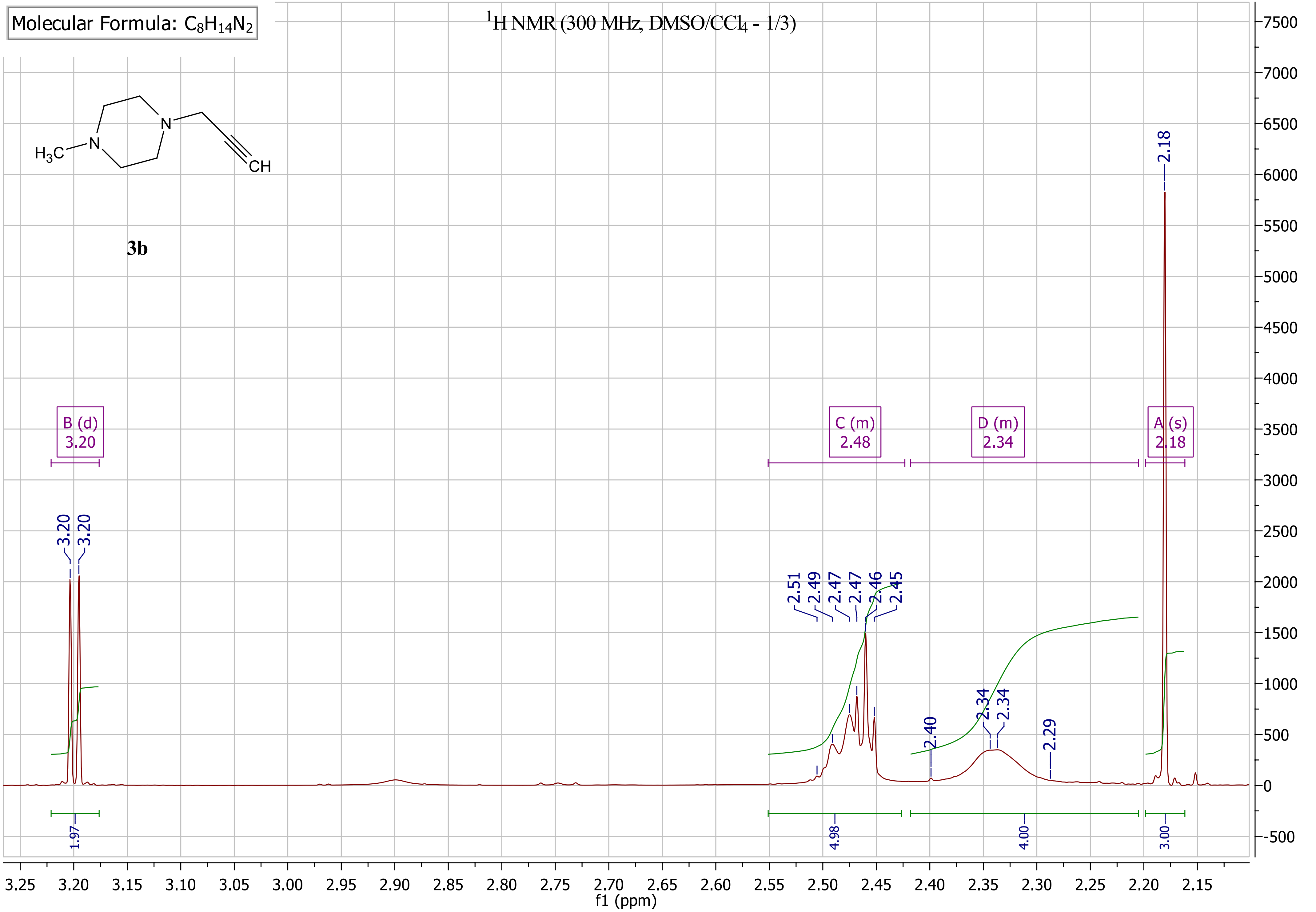
Molecular Formula: C8H14N2
1H NMR (300 MHz, DMSO/CCl4 - 1/3)
H3C
N
N
CH
3b
B (d) 3.20
C (m) 2.48
D (m) 2.34
A (s) 2.18
1.97
4.98
4.00
3.00
f1 (ppm)

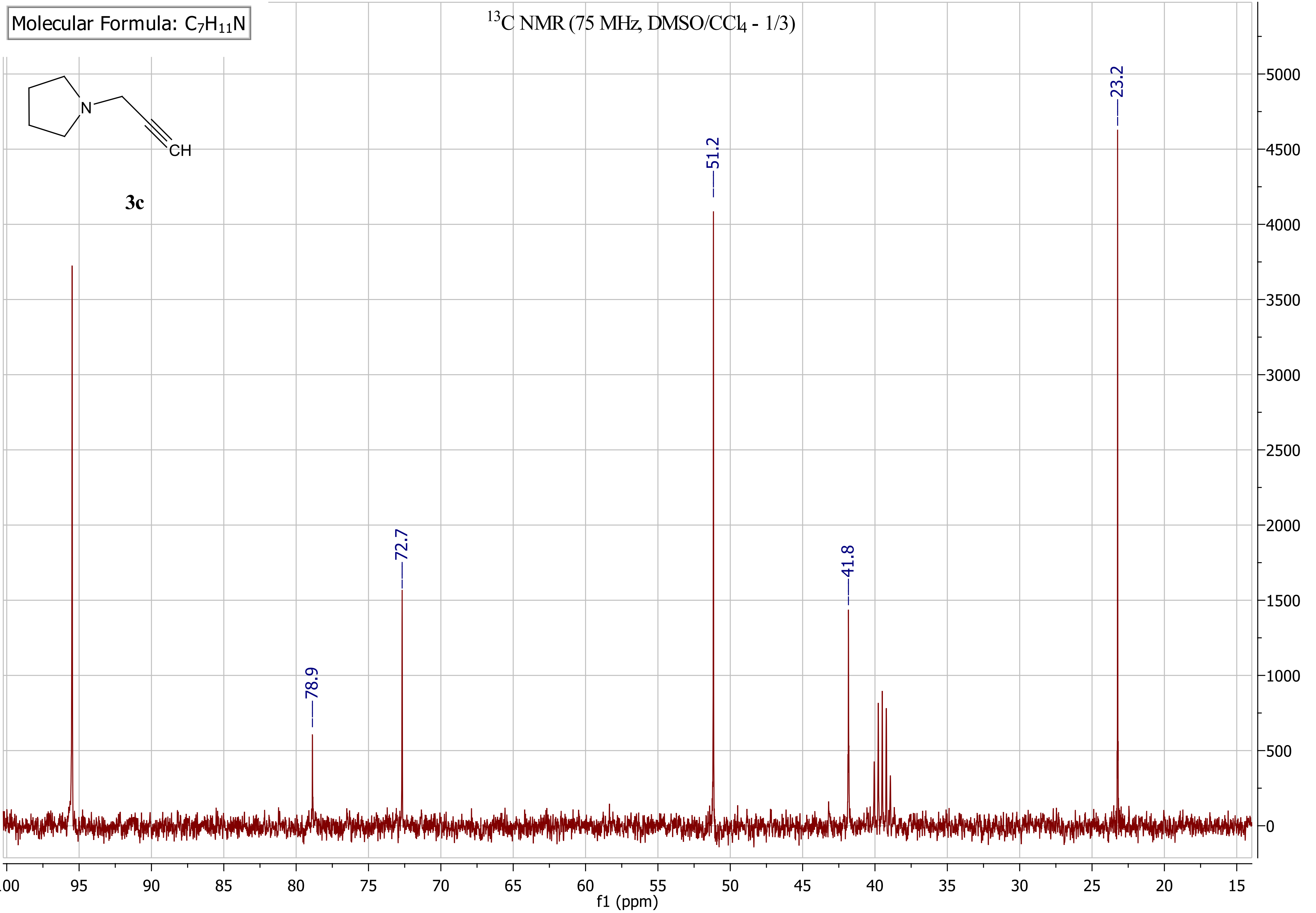
Molecular Formula: $C_7H_{11}N$
^{13}C NMR (75 MHz, DMSO/CCl_4 - 1/3)
N
CH
3c
78.9
72.7
51.2
41.8
23.2
5000
4500
4000
3500
3000
2500
2000
1500
1000
500
0
100
95
90
85
80
75
70
65
60
55
50
45
40
35
30
25
20
15
f1 (ppm)

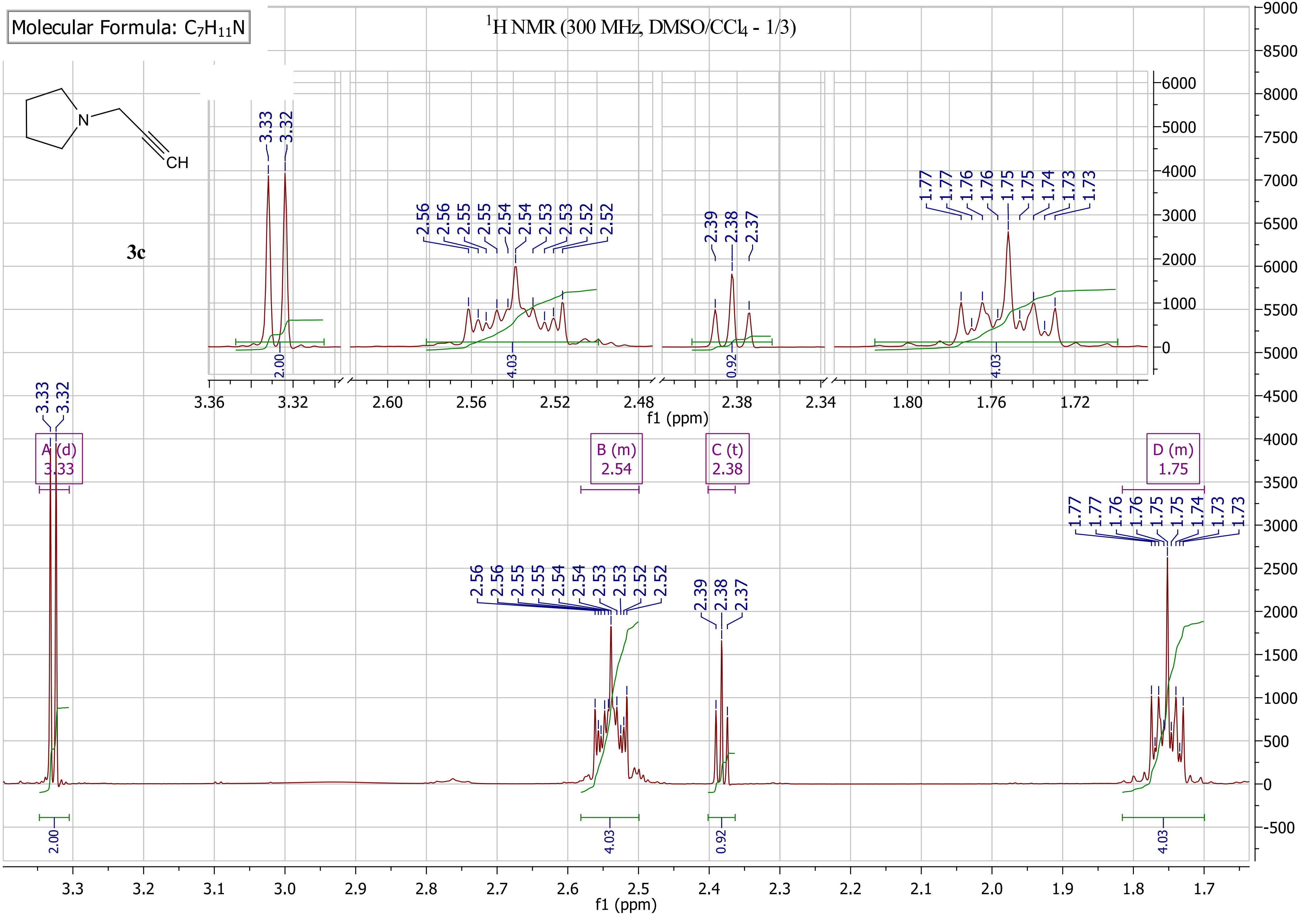

Molecular Formula: $C_7H_{11}N$
N
CH
3c
^{1}H NMR (300 MHz, DMSO/CCl$_4$ - 1/3)
3.33
3.32
2.00
2.56 2.56 2.55 2.55 2.54 2.54 2.53 2.53 2.52 2.52
4.03
2.39 2.38 2.37
0.92
1.77 1.77 1.76 1.76 1.75 1.75 1.74 1.73 1.73
4.03
A (d)
3.33
B (m)
2.54
C (t)
2.38
D (m)
1.75
f1 (ppm)
f1 (ppm)

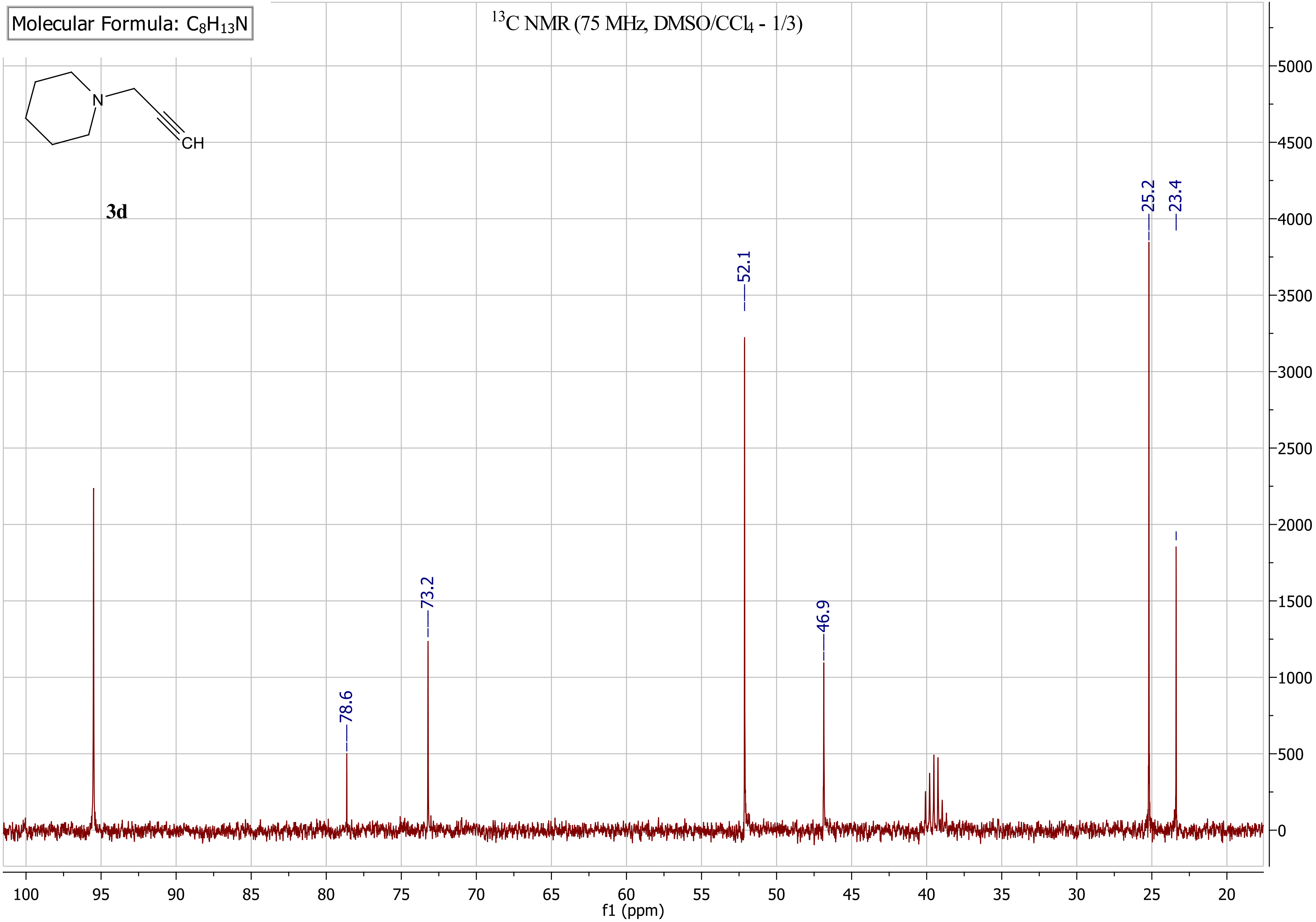

Molecular Formula: $C_8H_{13}N$
^{13}C NMR (75 MHz, DMSO/CCl_4 - 1/3)
N
CH
3d
78.6
73.2
52.1
46.9
25.2
23.4
f1 (ppm)

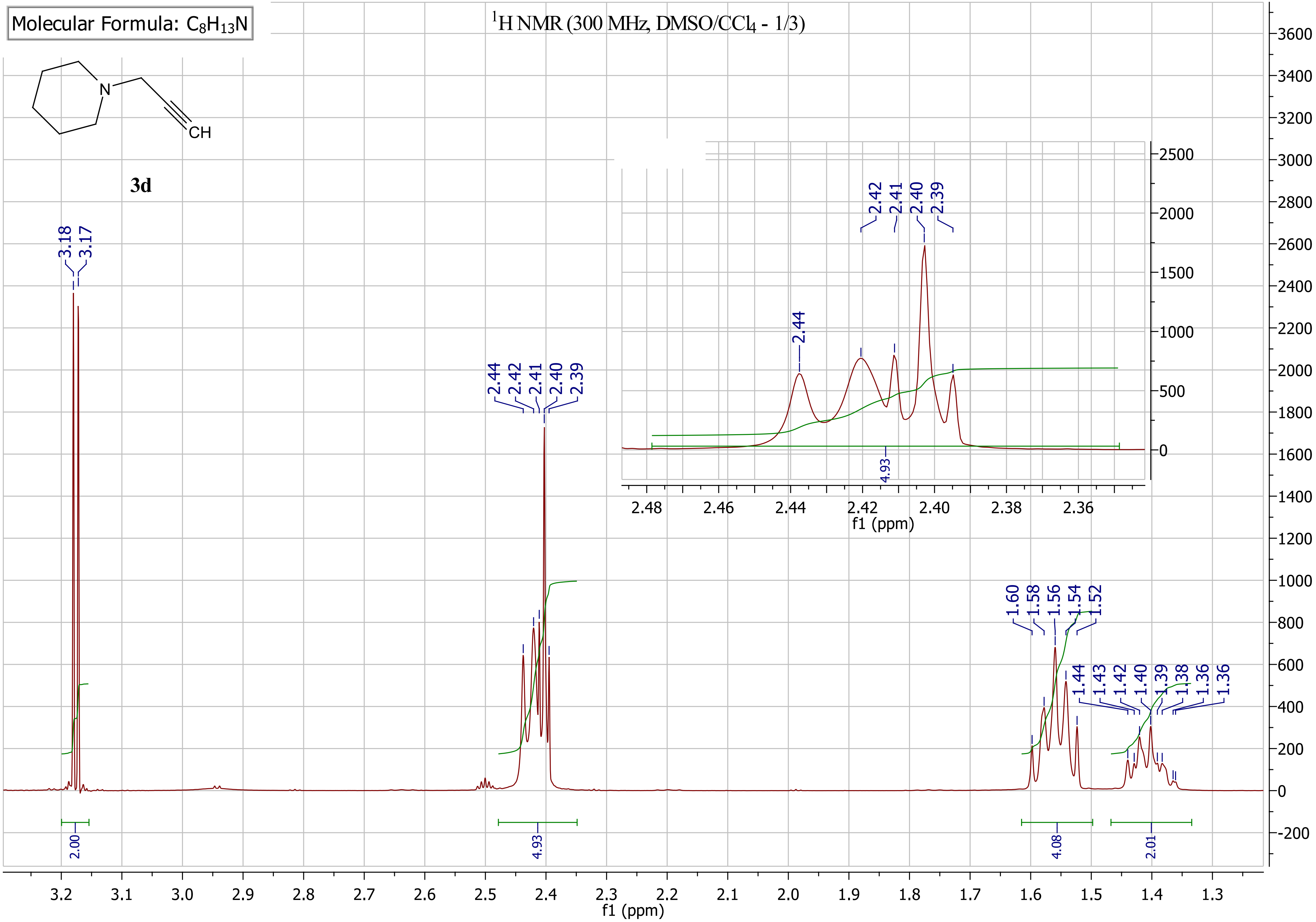

Molecular Formula: C8H13N
1H NMR (300 MHz, DMSO/CCl4 - 1/3)
N
CH
3d
3.18
3.17
2.44
2.42
2.41
2.40
2.39
1.60
1.58
1.56
1.54
1.52
1.44
1.43
1.42
1.40
1.39
1.38
1.36
1.36
2.00
4.93
4.08
2.01
f1 (ppm)

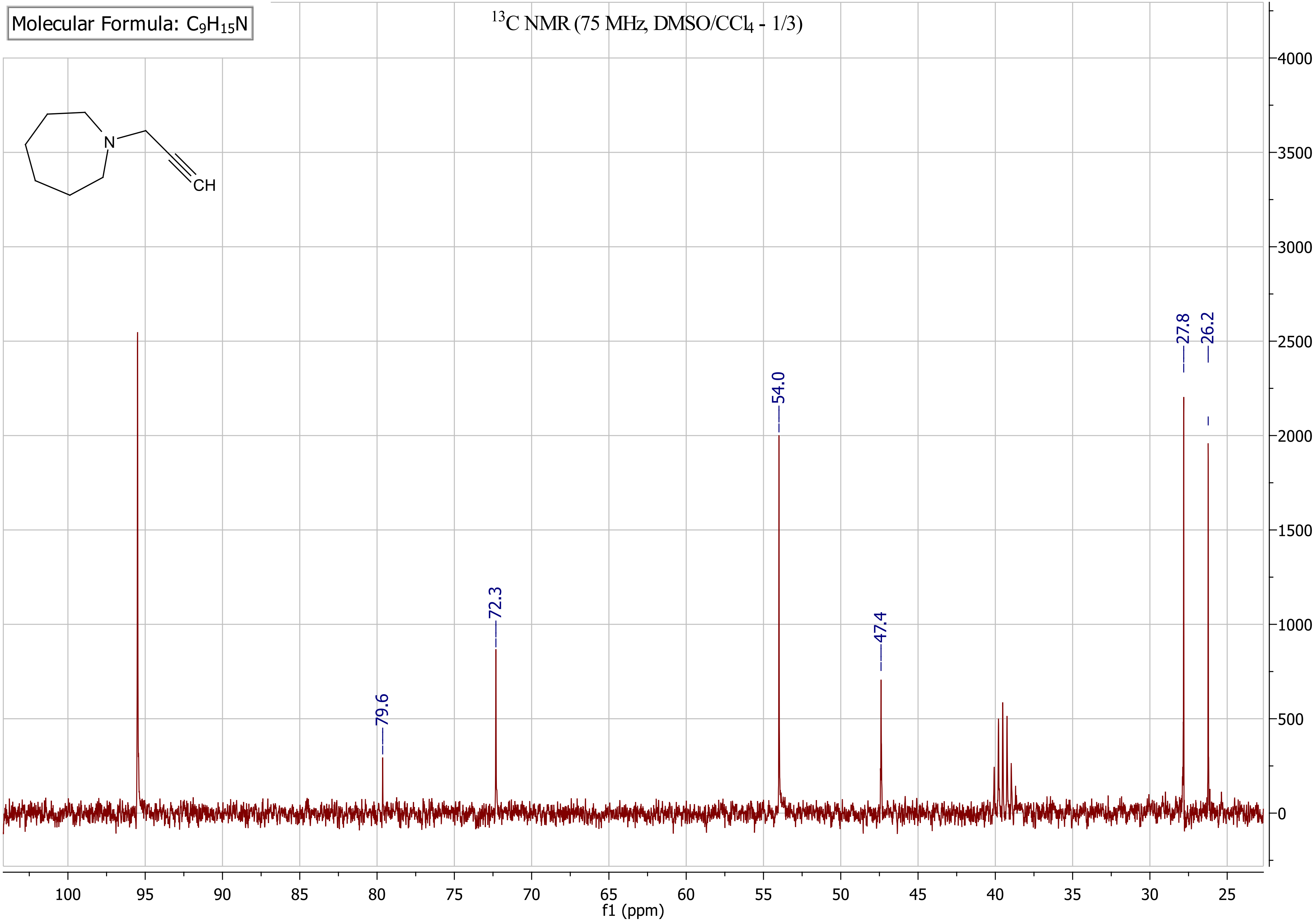
Molecular Formula: C9H15N
13C NMR (75 MHz, DMSO/CCl4 - 1/3)
N
CH
79.6
72.3
54.0
47.4
27.8
26.2
4000
3500
3000
2500
2000
1500
1000
500
0
100
95
90
85
80
75
70
65
60
55
50
45
40
35
30
25
f1 (ppm)

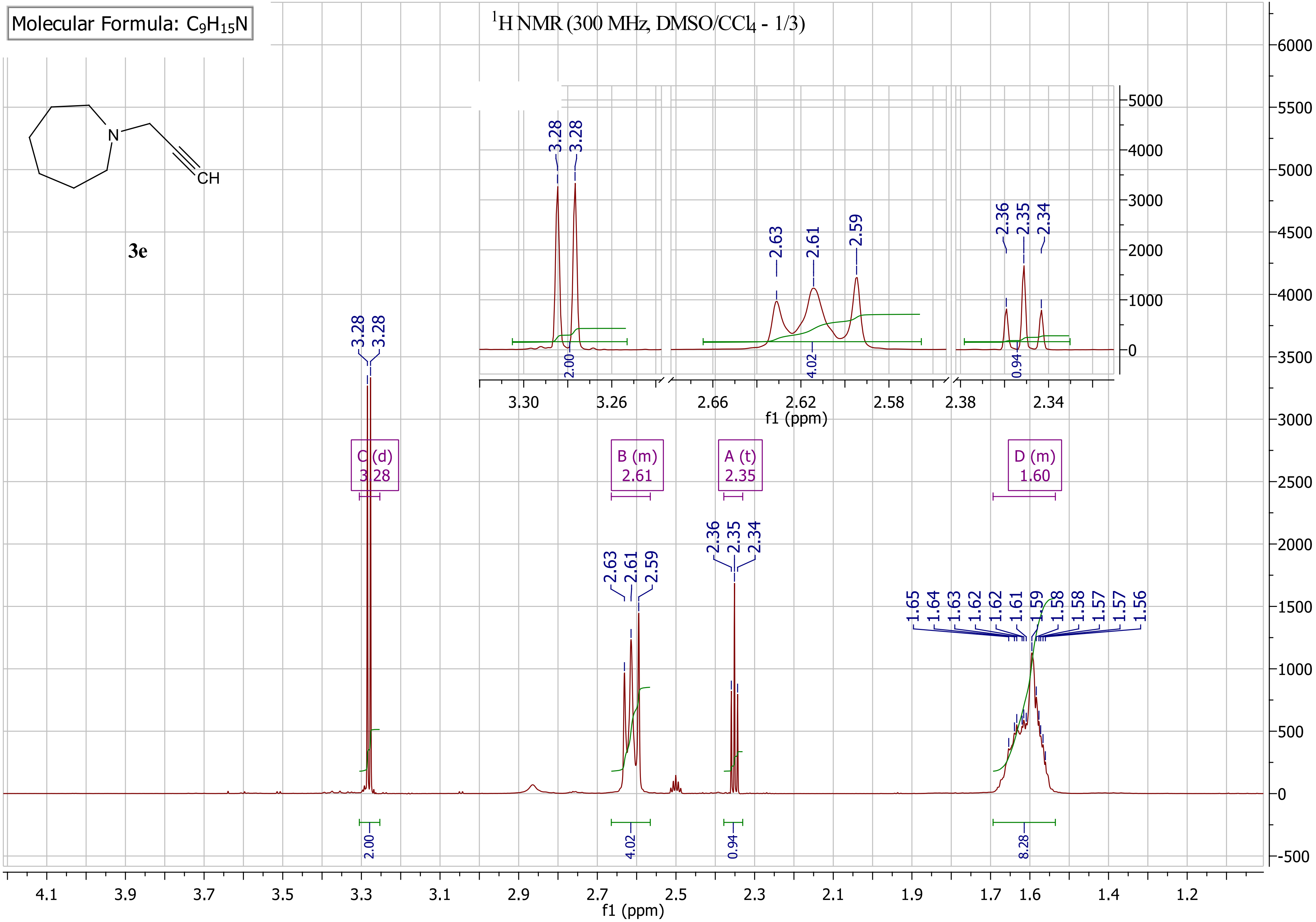

Molecular Formula: $C_9H_{15}N$
^{1}H NMR (300 MHz, DMSO/CCl_4 - 1/3)
N
CH
3e
C (d) 3.28
B (m) 2.61
A (t) 2.35
D (m) 1.60
3.28
3.28
2.63
2.61
2.59
2.36
2.35
2.34
1.65
1.64
1.63
1.62
1.62
1.61
1.59
1.58
1.58
1.57
1.57
1.56
2.00
4.02
0.94
8.28
f1 (ppm)

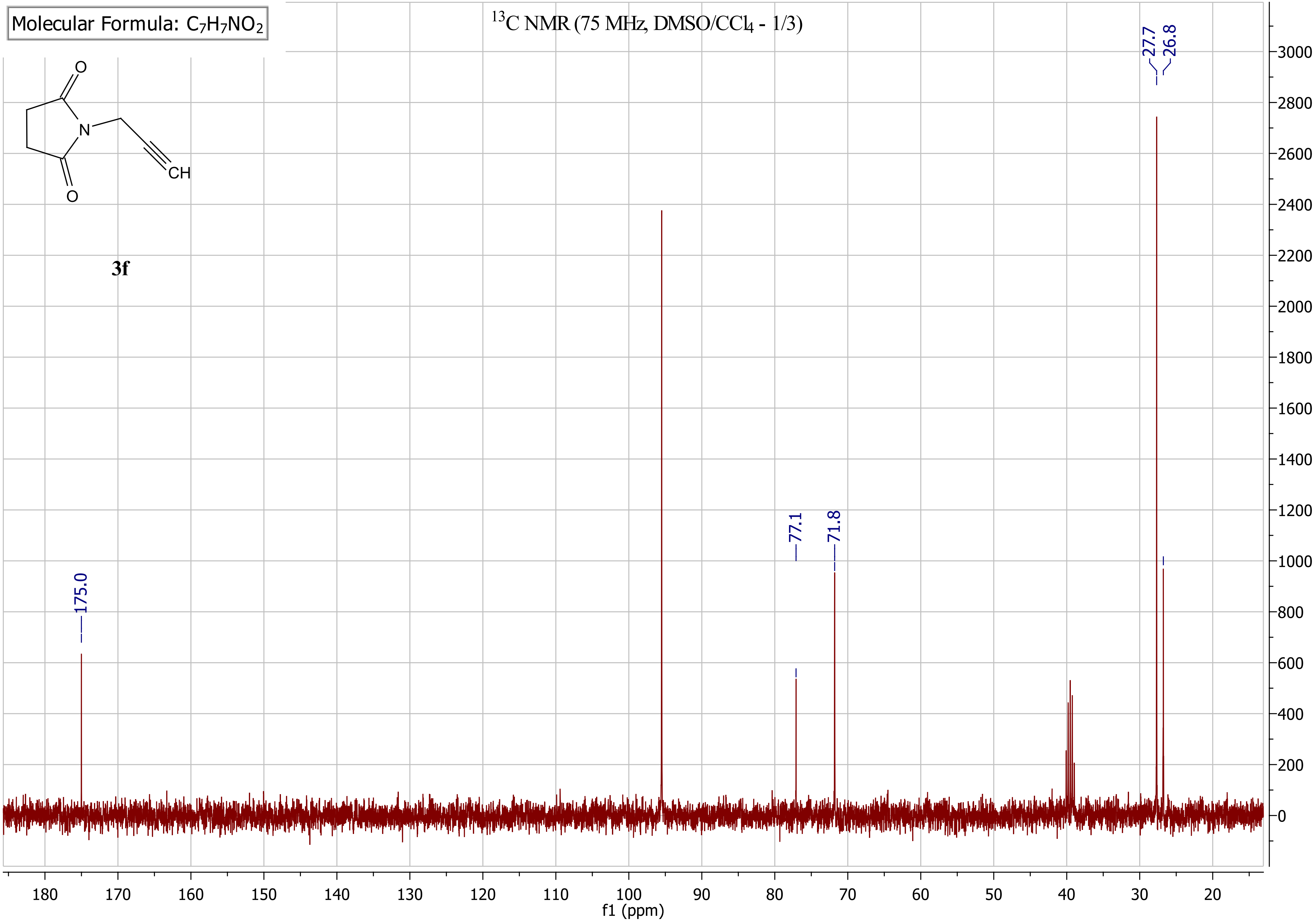

Molecular Formula: $C_7H_7NO_2$
^{13}C NMR (75 MHz, DMSO/CCl$_4$ - 1/3)
O
N
CH
O
3f
175.0
77.1
71.8
27.7
26.8
f1 (ppm)
180
170
160
150
140
130
120
110
100
90
80
70
60
50
40
30
20
3000
2800
2600
2400
2200
2000
1800
1600
1400
1200
1000
800
600
400
200
0

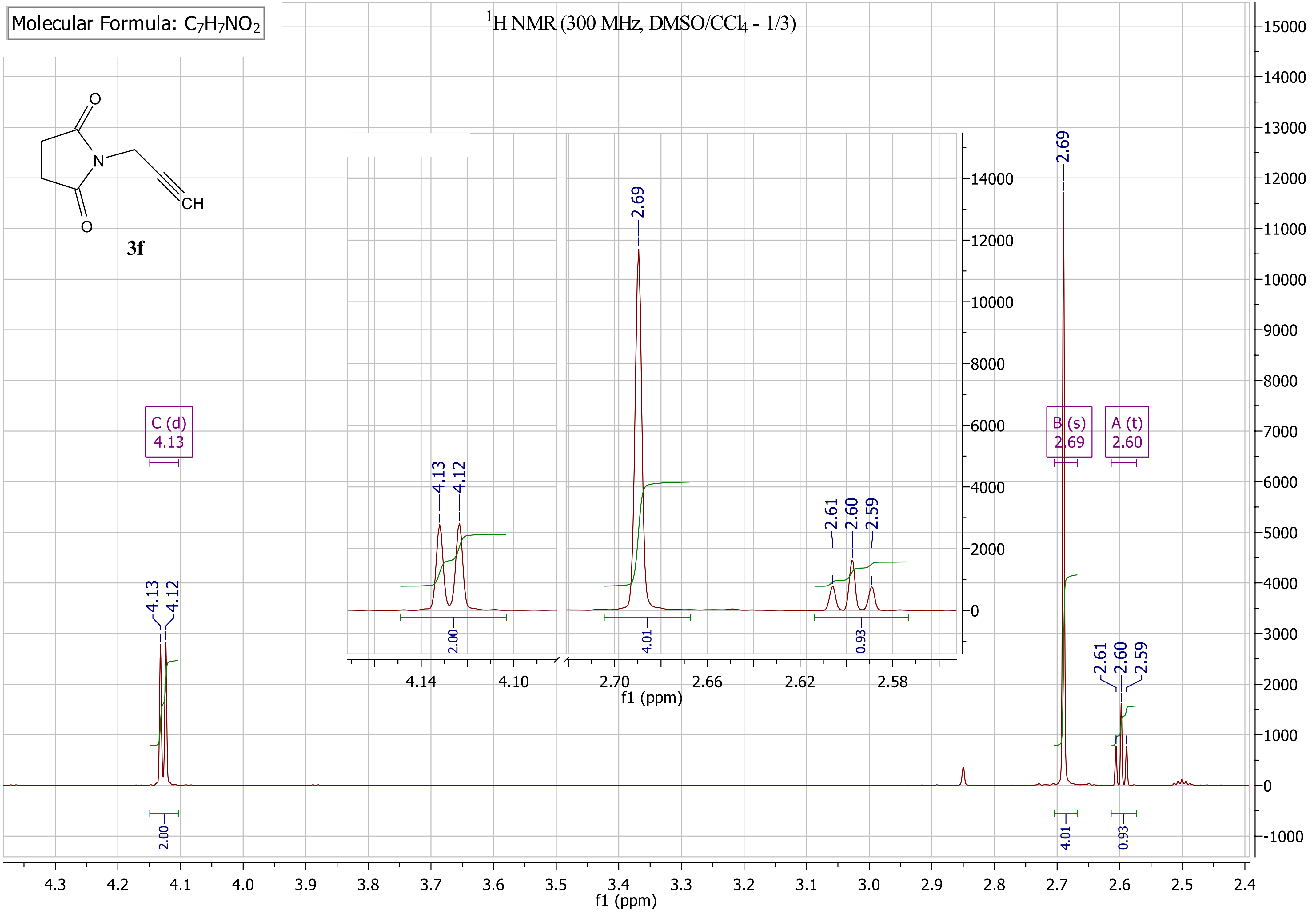

Molecular Formula: C7H7NO2
1H NMR (300 MHz, DMSO/CCl4 - 1/3)
O
N
CH
O
3f
C (d)
4.13
B (s)
2.69
A (t)
2.60
4.13
4.12
2.69
2.61
2.60
2.59
2.00
4.01
0.93
f1 (ppm)

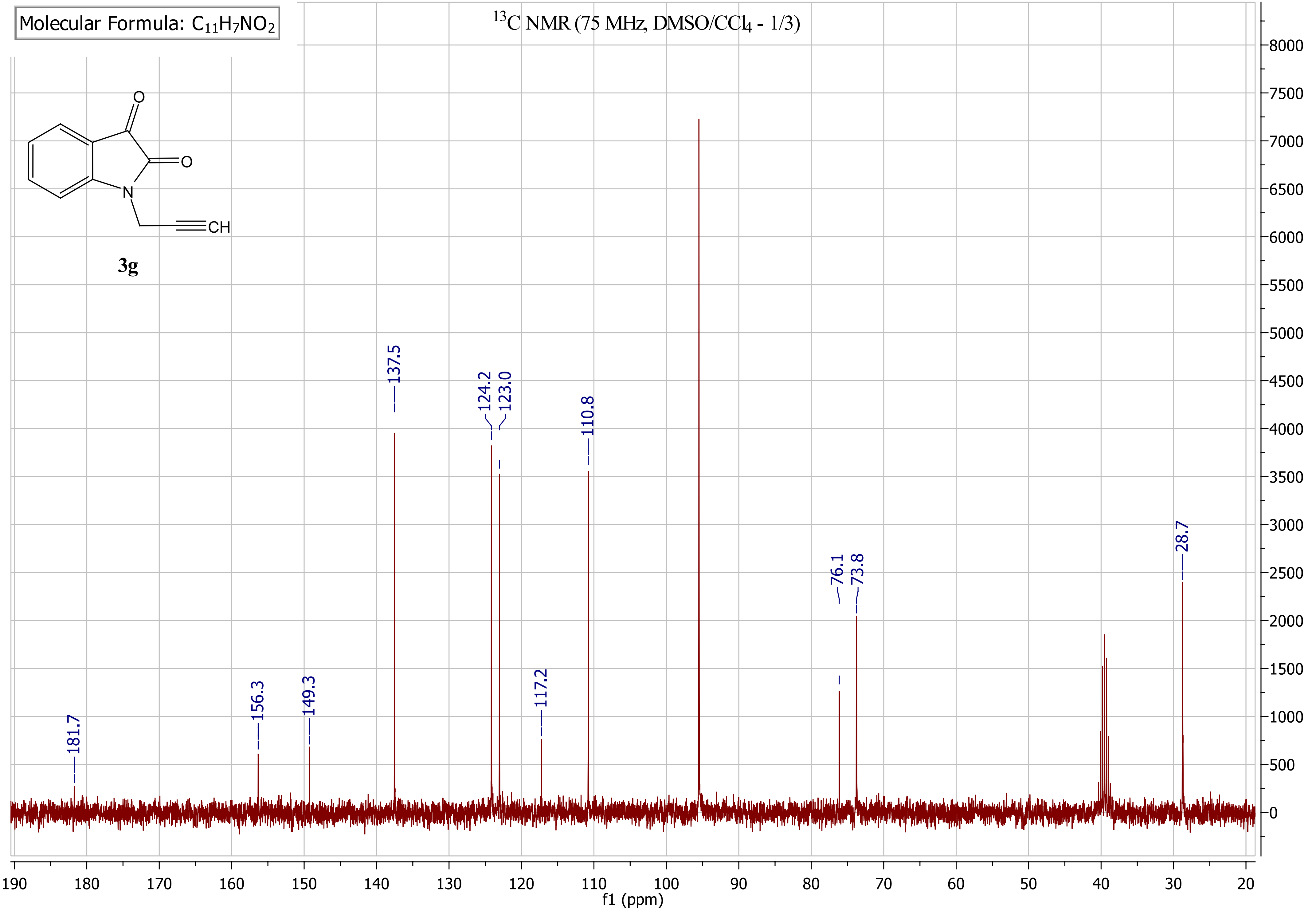
Molecular Formula: $C_{11}H_7NO_2$
^{13}C NMR (75 MHz, DMSO/CCl_4 - 1/3)
O
O
N
CH
3g
181.7
156.3
149.3
137.5
124.2
123.0
117.2
110.8
76.1
73.8
28.7
f1 (ppm)
190 180 170 160 150 140 130 120 110 100 90 80 70 60 50 40 30 20
8000 7500 7000 6500 6000 5500 5000 4500 4000 3500 3000 2500 2000 1500 1000 500 0

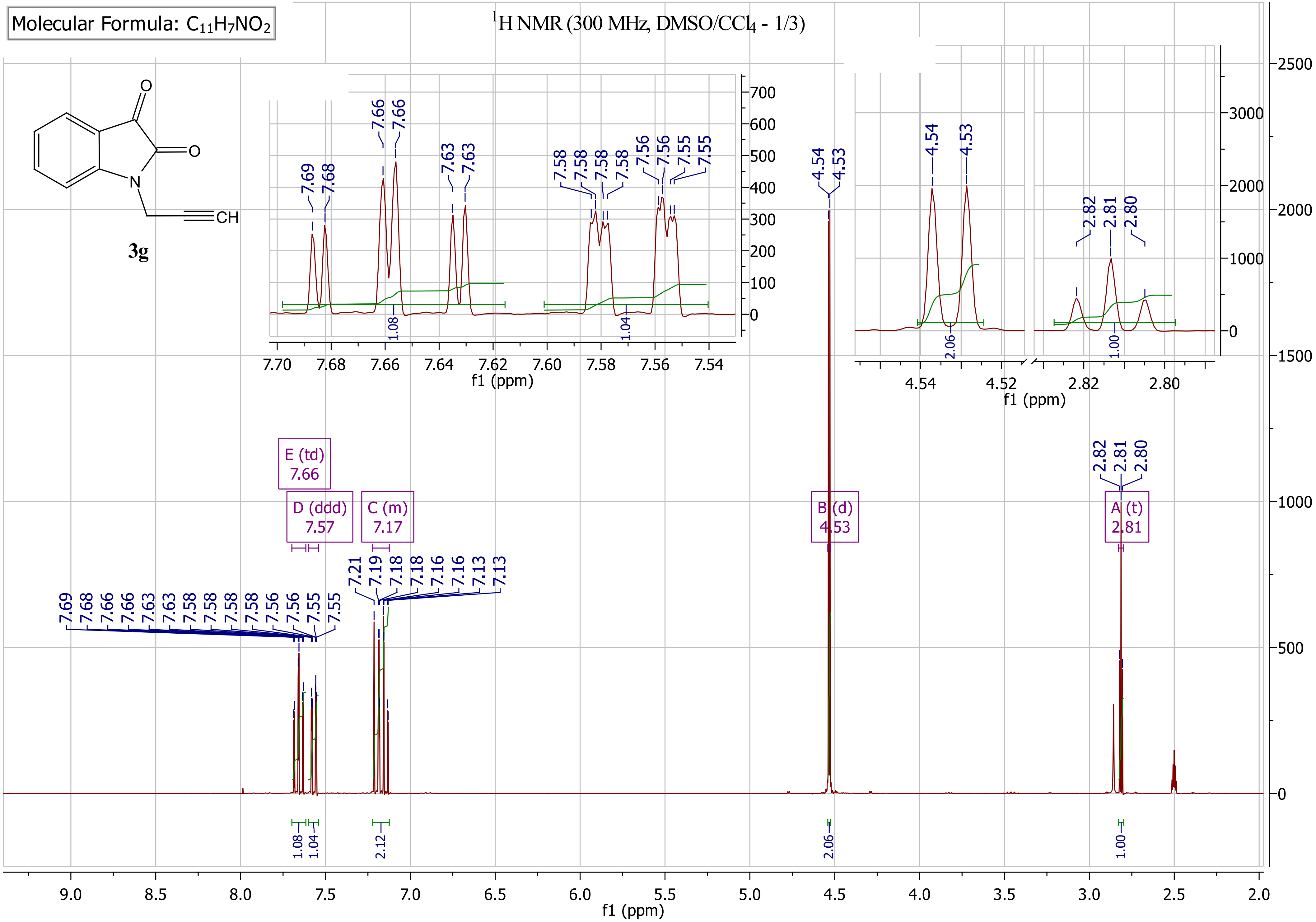

Molecular Formula: C11H7NO2
1H NMR (300 MHz, DMSO/CCl4 - 1/3)
3g
O
O
N
CH
E (td) 7.66
D (ddd) 7.57
C (m) 7.17
B (d) 4.53
A (t) 2.81
7.69 7.68 7.66 7.66 7.63 7.63 7.58 7.58 7.58 7.58 7.56 7.56 7.55 7.55
7.21 7.19 7.18 7.18 7.16 7.16 7.13 7.13
4.54 4.53
2.82 2.81 2.80
1.08 1.04 2.12 2.06 1.00
f1 (ppm)

Molecular Formula: $C_{11}H_7NO_2$

^{13}C NMR (75 MHz, DMSO/CCl_4 - 1/3)

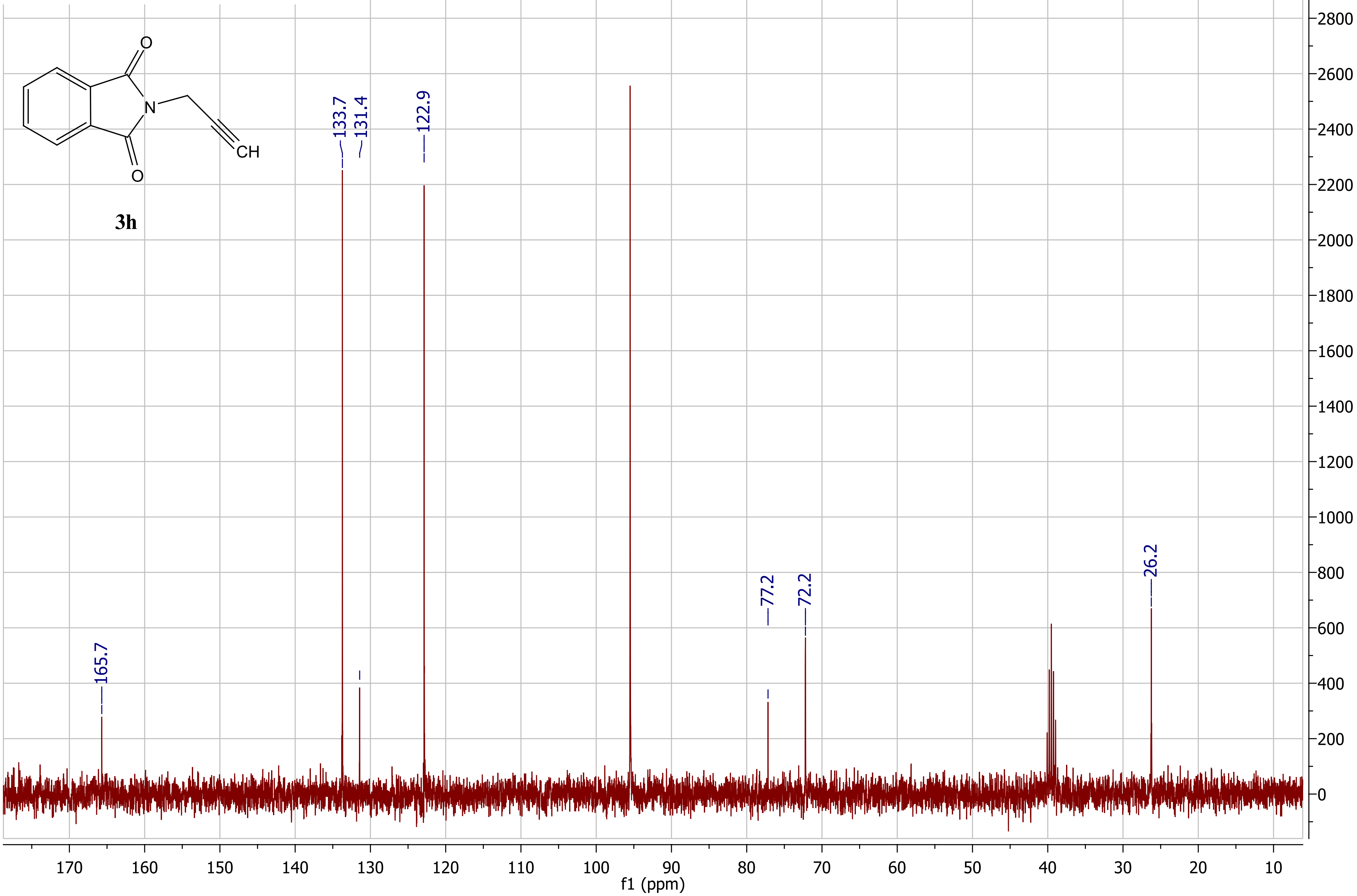

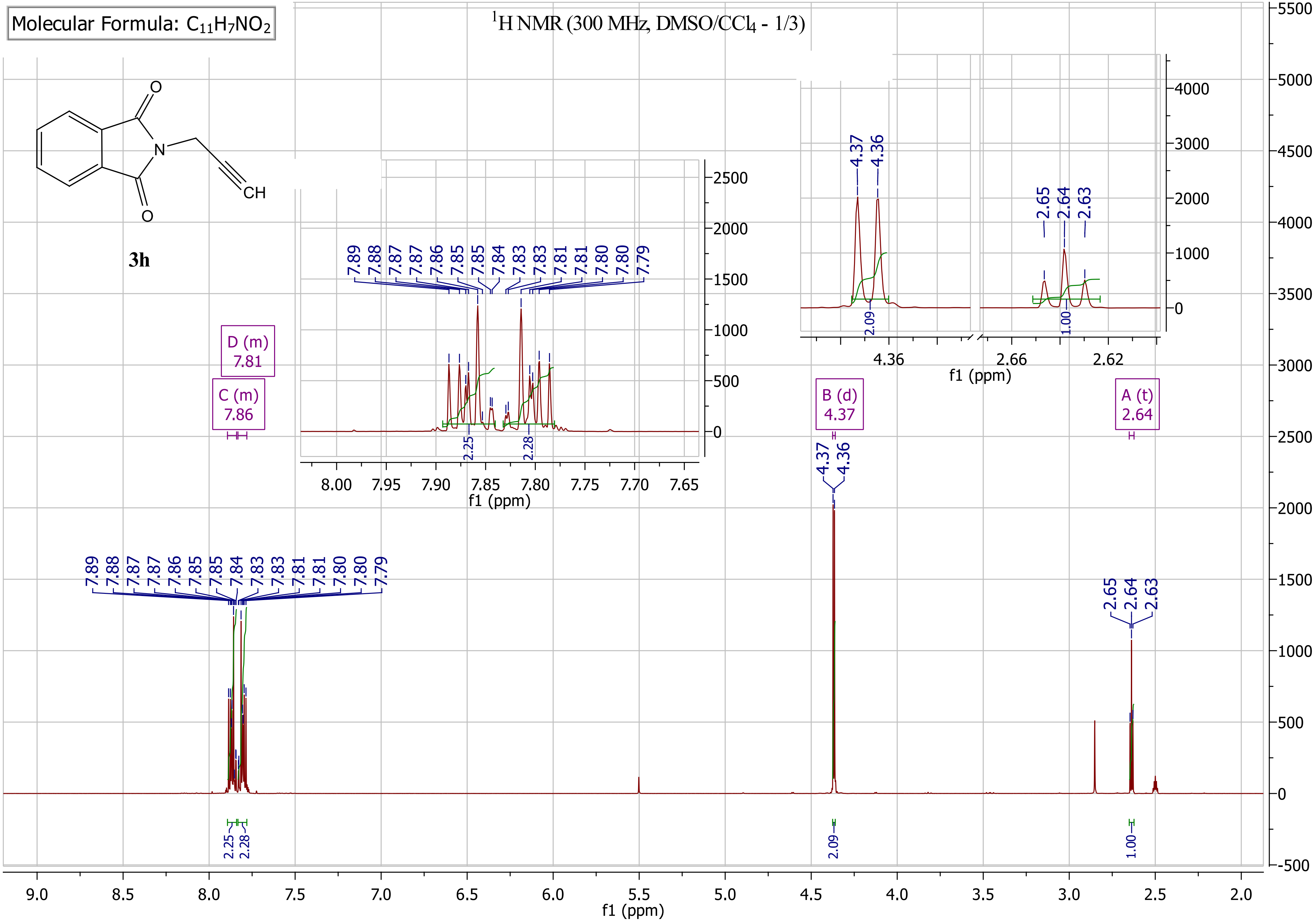

Molecular Formula: C11H7NO2
1H NMR (300 MHz, DMSO/CCl4 - 1/3)
3h
O
N
CH
O
D (m)
7.81
C (m)
7.86
B (d)
4.37
A (t)
2.64
7.89 7.88 7.87 7.87 7.86 7.85 7.85 7.84 7.83 7.83 7.81 7.81 7.80 7.80 7.79
4.37 4.36
2.65 2.64 2.63
2.25 2.28 2.09 1.00
f1 (ppm)

Molecular Formula: $C_9H_7N_3$

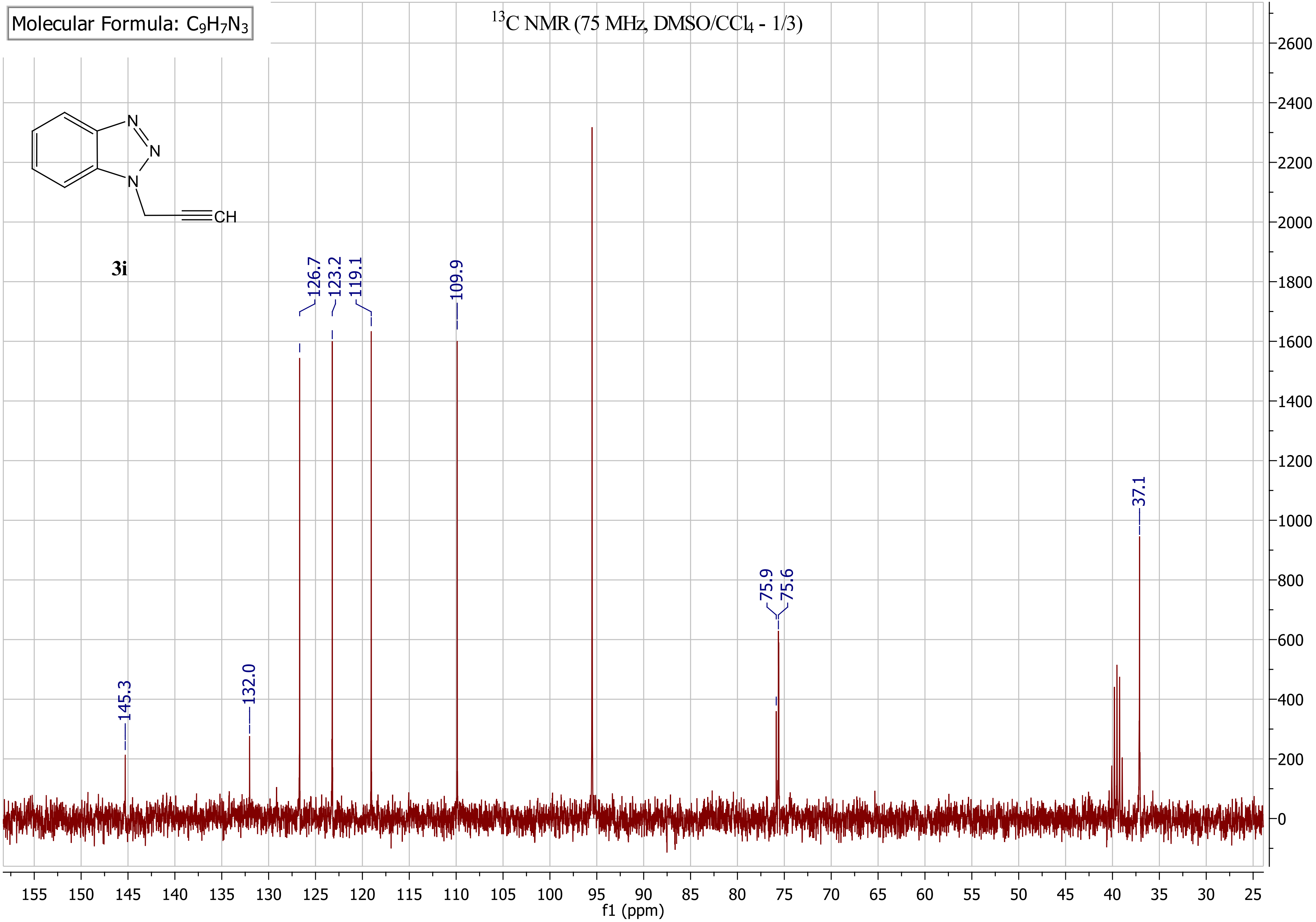

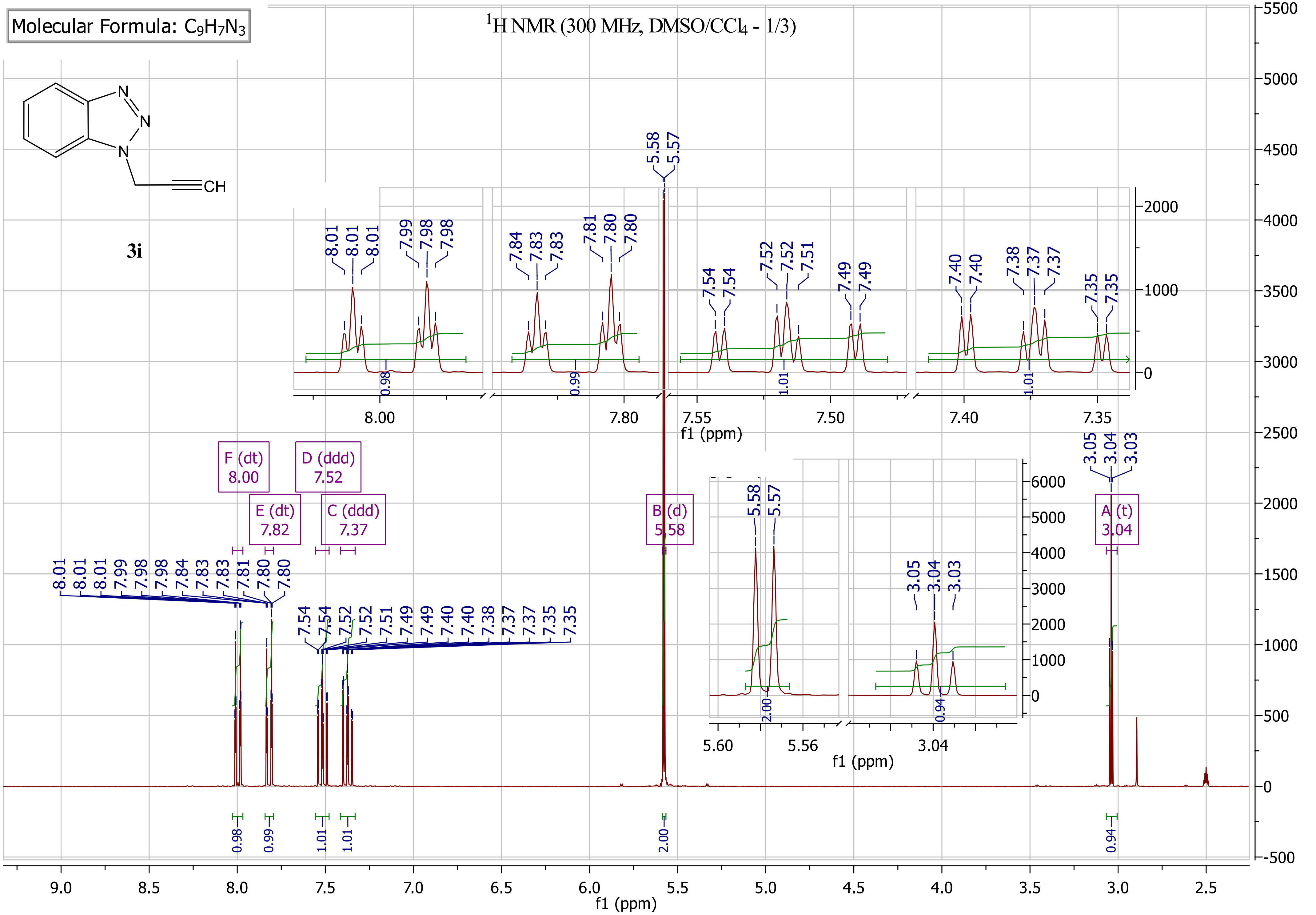

Molecular Formula: $C_9H_7N_3$
^{1}H NMR (300 MHz, DMSO/CCl_4 - 1/3)
N
N
N
CH
3i
F (dt) 8.00
E (dt) 7.82
D (ddd) 7.52
C (ddd) 7.37
B (d) 5.58
A (t) 3.04
8.01 8.01 8.01 7.99 7.98 7.98 7.84 7.83 7.83 7.81 7.80 7.80
7.54 7.54 7.52 7.52 7.51 7.49 7.49 7.40 7.40 7.38 7.37 7.37 7.35 7.35
5.58 5.57
3.05 3.04 3.03
0.98 0.99 1.01 1.01 2.00 0.94
f1 (ppm)

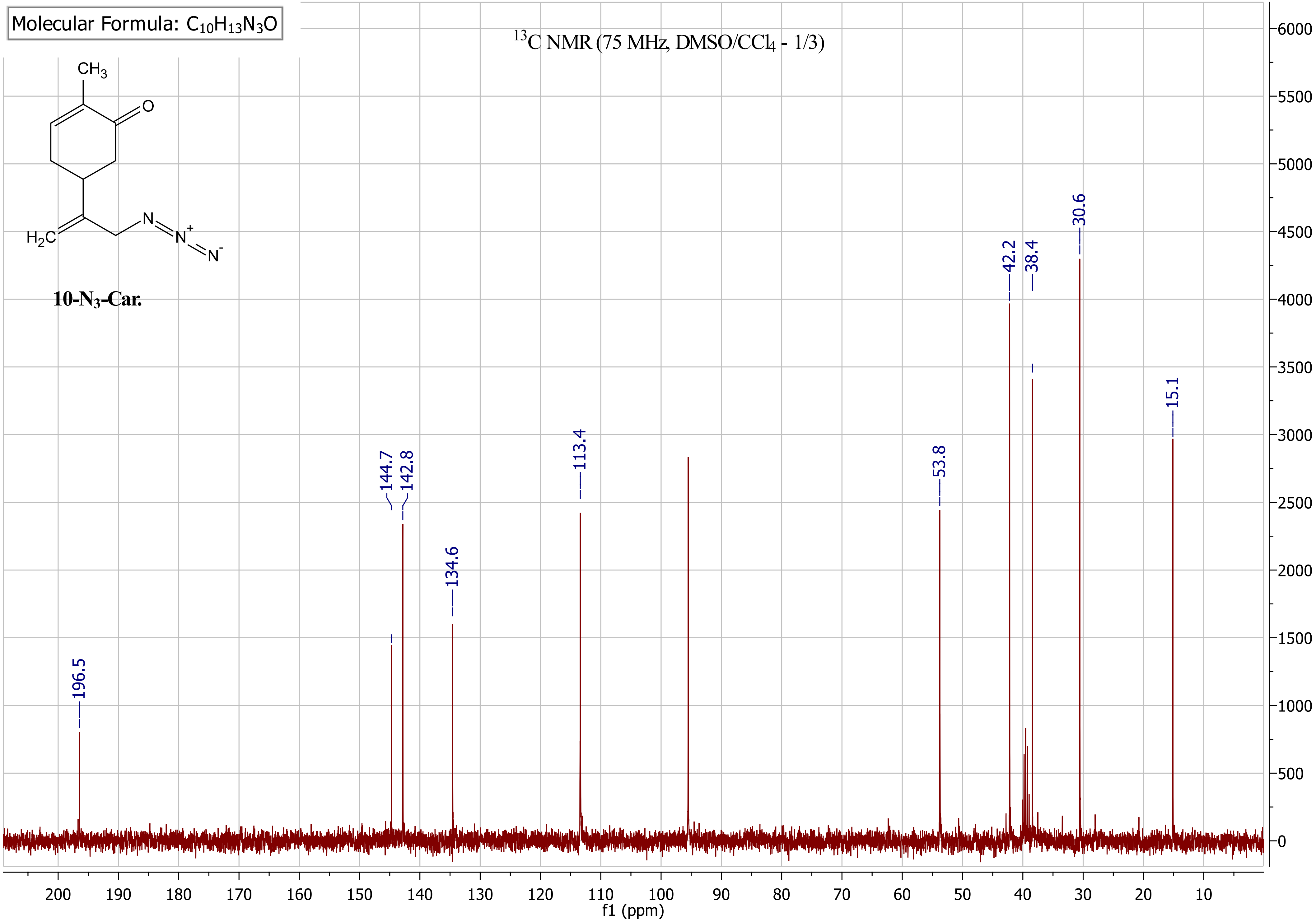
Molecular Formula: $C_{10}H_{13}N_3O$
^{13}C NMR (75 MHz, DMSO/CCl_4 - 1/3)
CH_3
O
H_2C
N
N^+
N^-
10-N_3-Car.
196.5
144.7
142.8
134.6
113.4
53.8
42.2
38.4
30.6
15.1
f1 (ppm)

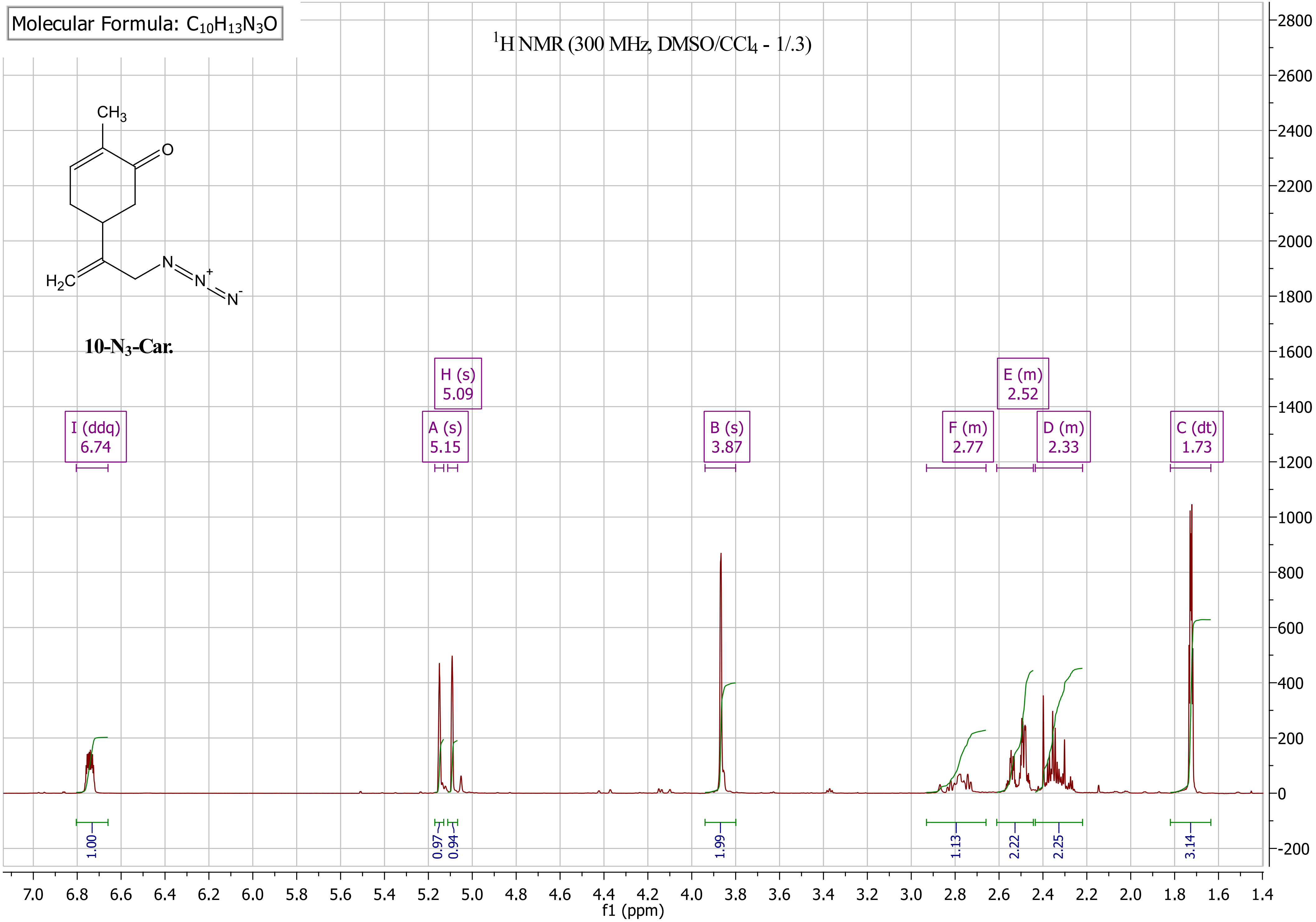

Molecular Formula: C10H13N3O
1H NMR (300 MHz, DMSO/CCl4 - 1/.3)
CH3
O
H2C
N
N+
N-
10-N3-Car.
I (ddq) 6.74
A (s) 5.15
H (s) 5.09
B (s) 3.87
F (m) 2.77
E (m) 2.52
D (m) 2.33
C (dt) 1.73
1.00
0.97
0.94
1.99
1.13
2.22
2.25
3.14
f1 (ppm)

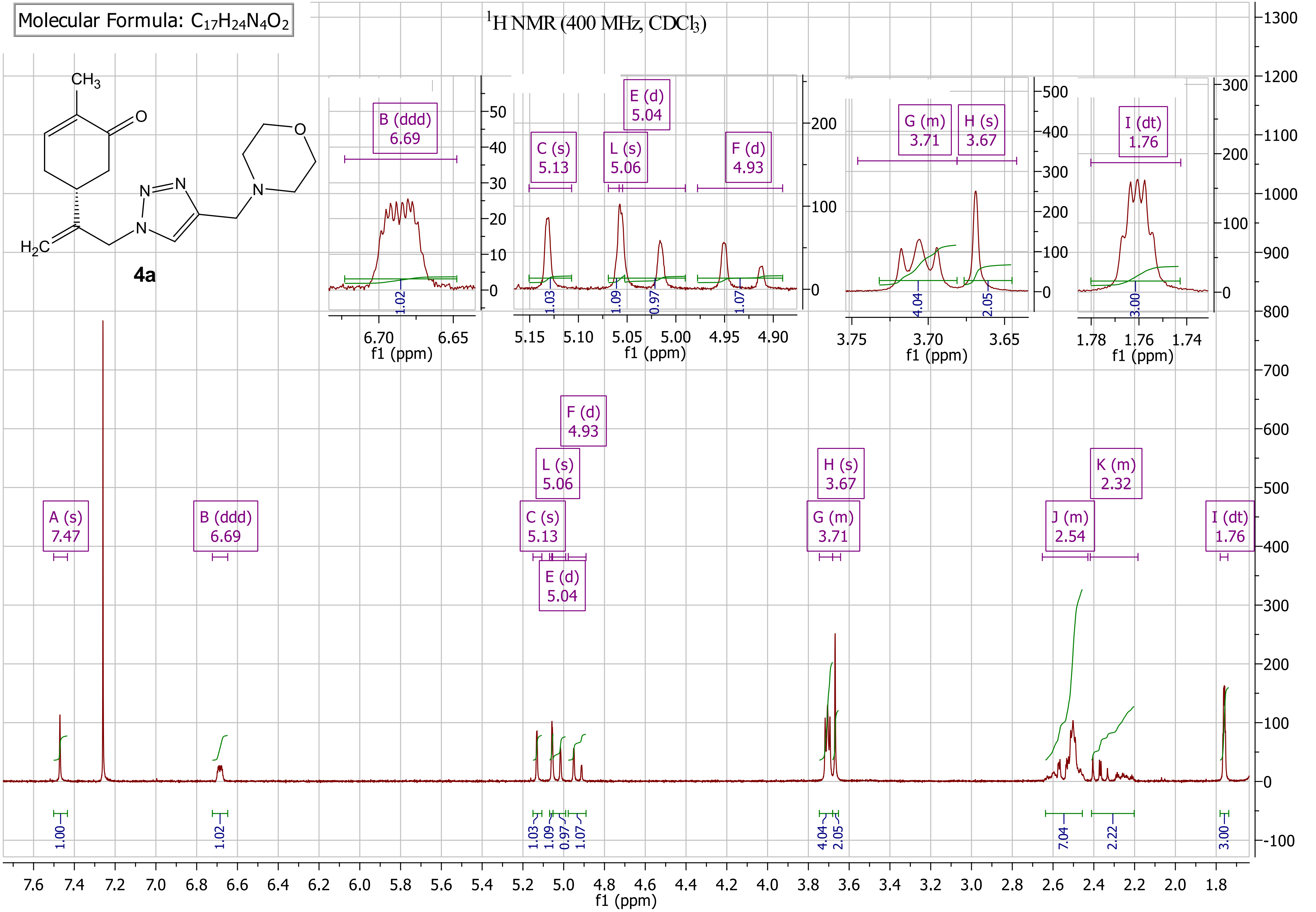

Molecular Formula: $C_{17}H_{24}N_4O_2$
^{1}H NMR (400 MHz, $CDCl_3$)
4a
A (s) 7.47
B (ddd) 6.69
C (s) 5.13
L (s) 5.06
E (d) 5.04
F (d) 4.93
G (m) 3.71
H (s) 3.67
J (m) 2.54
K (m) 2.32
I (dt) 1.76
1.00
1.02
1.03
1.09
0.97
1.07
4.04
2.05
7.04
2.22
3.00
f1 (ppm)

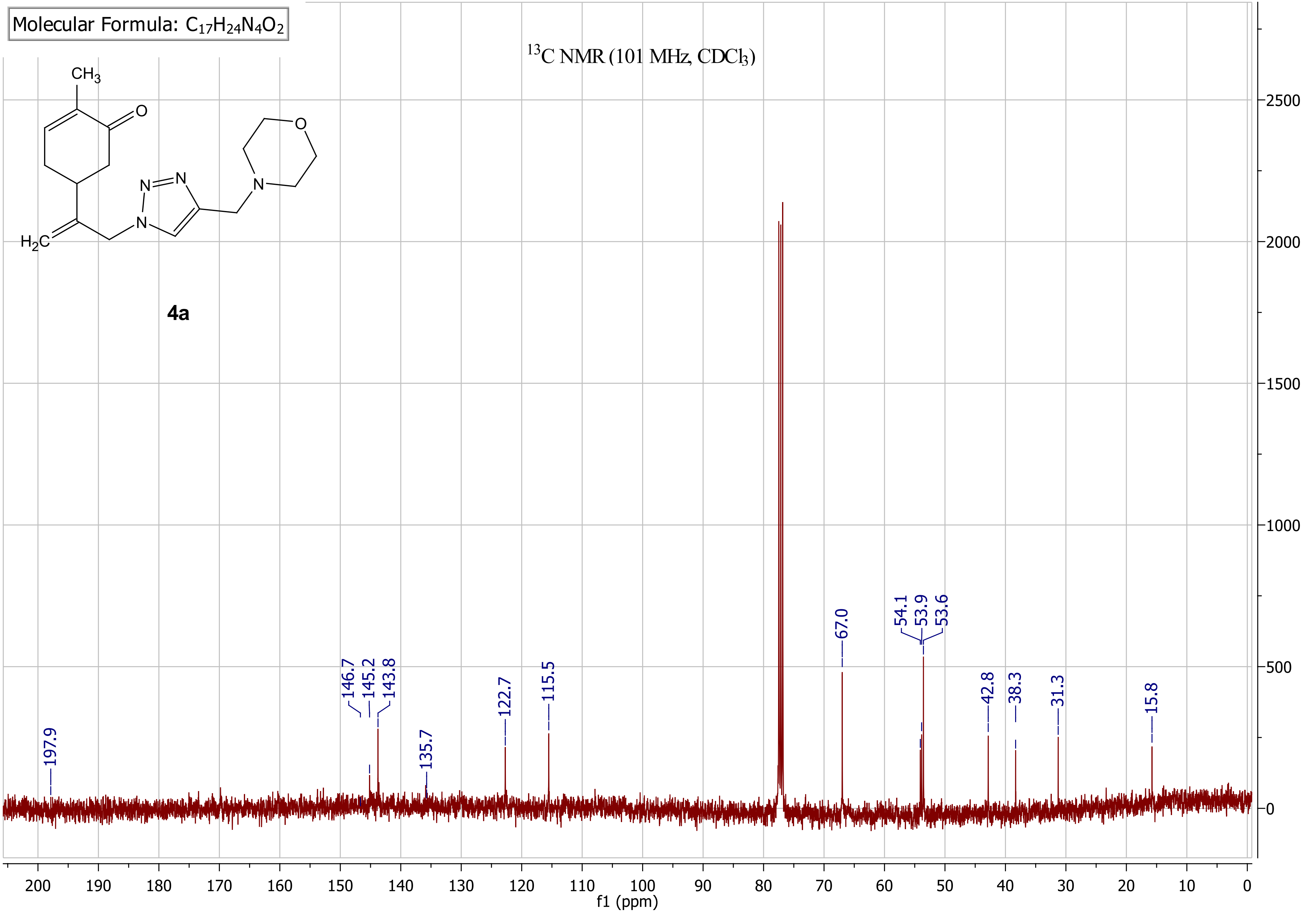

Molecular Formula: $C_{17}H_{24}N_4O_2$
4a
^{13}C NMR (101 MHz, $CDCl_3$)
197.9
146.7
145.2
143.8
135.7
122.7
115.5
67.0
54.1
53.9
53.6
42.8
38.3
31.3
15.8
f1 (ppm)

Molecular Formula: $C_{18}H_{27}N_5O$

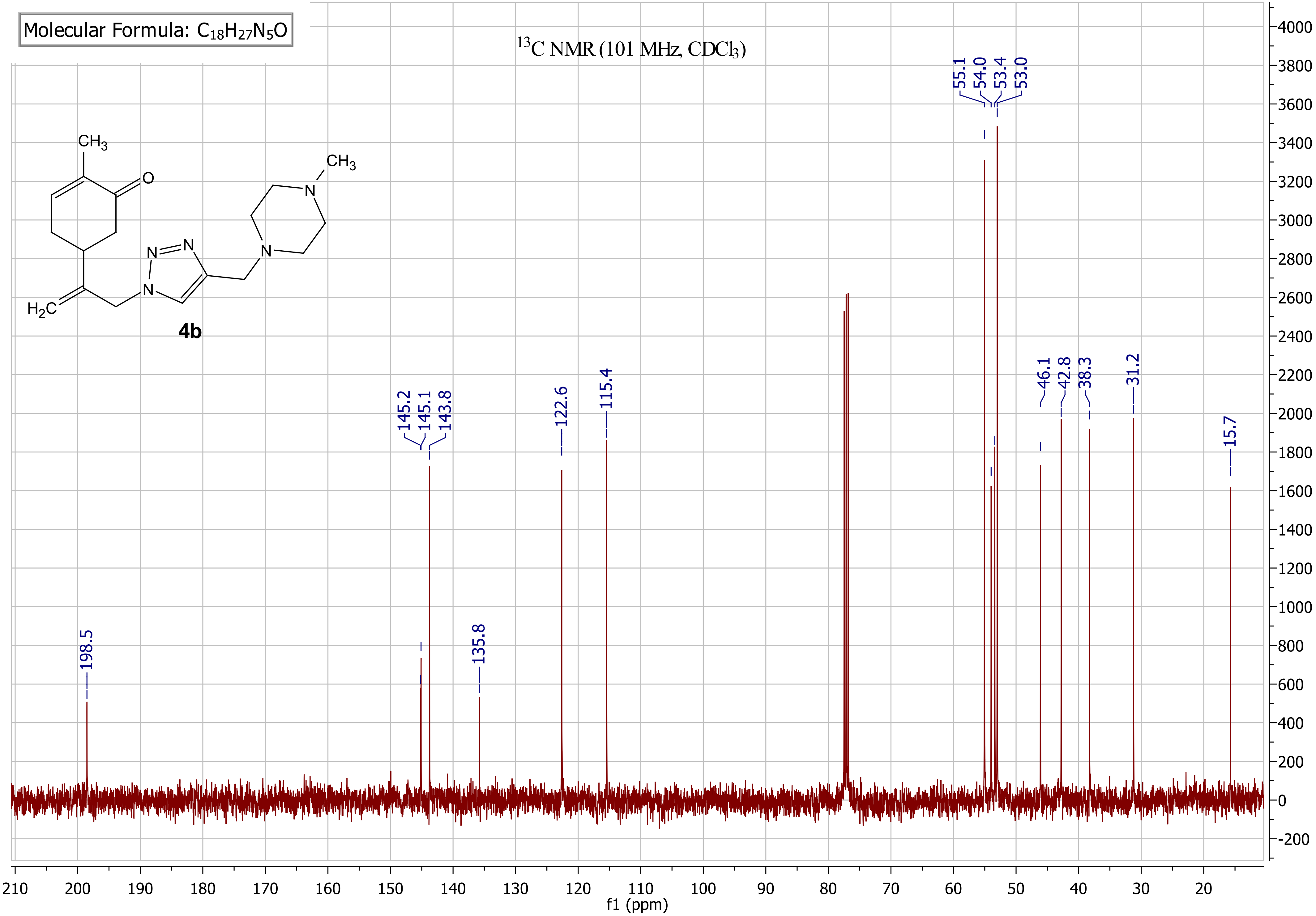

Molecular Formula: $C_{18}H_{27}N_5O$

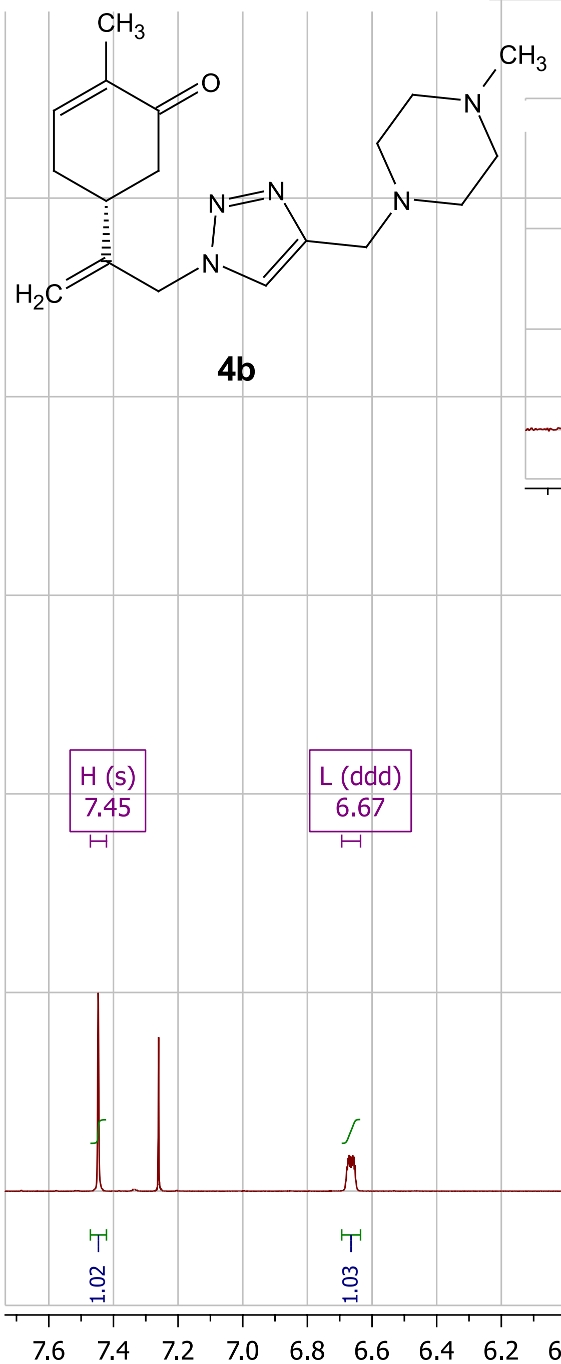

4b

^1H NMR (400 MHz, $CDCl_3$)

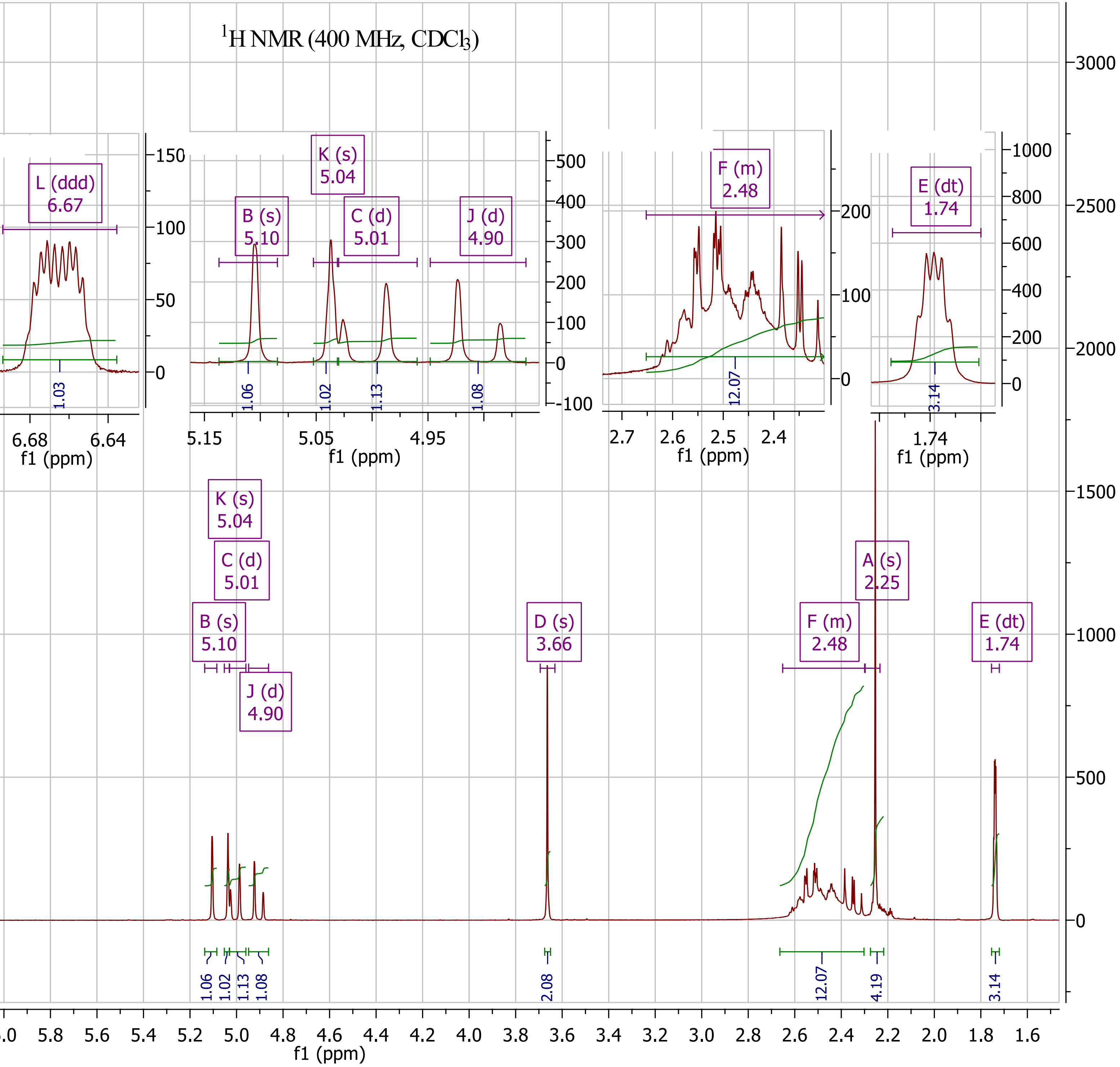

Molecular Formula: $C_{17}H_{24}N_4O$

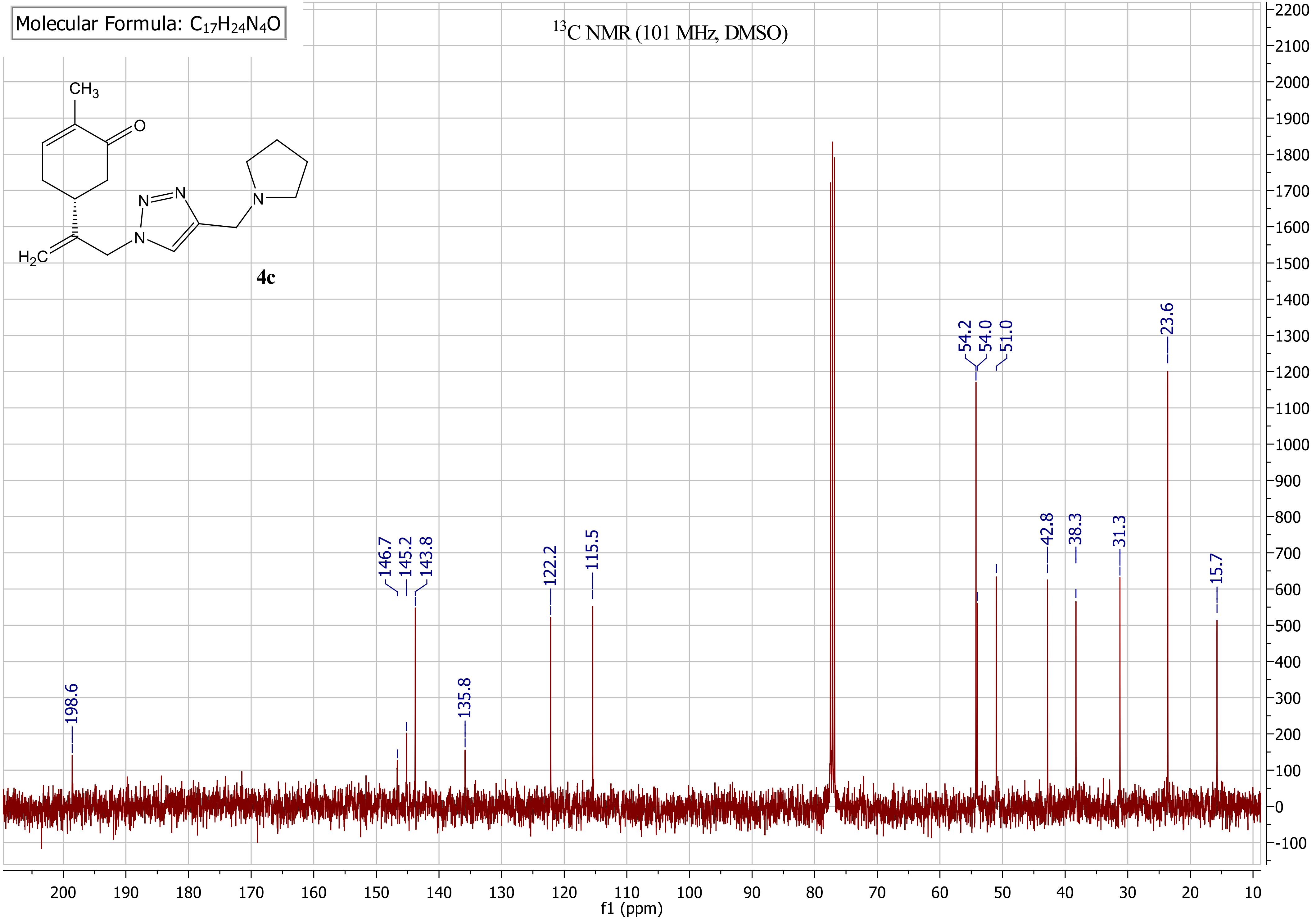

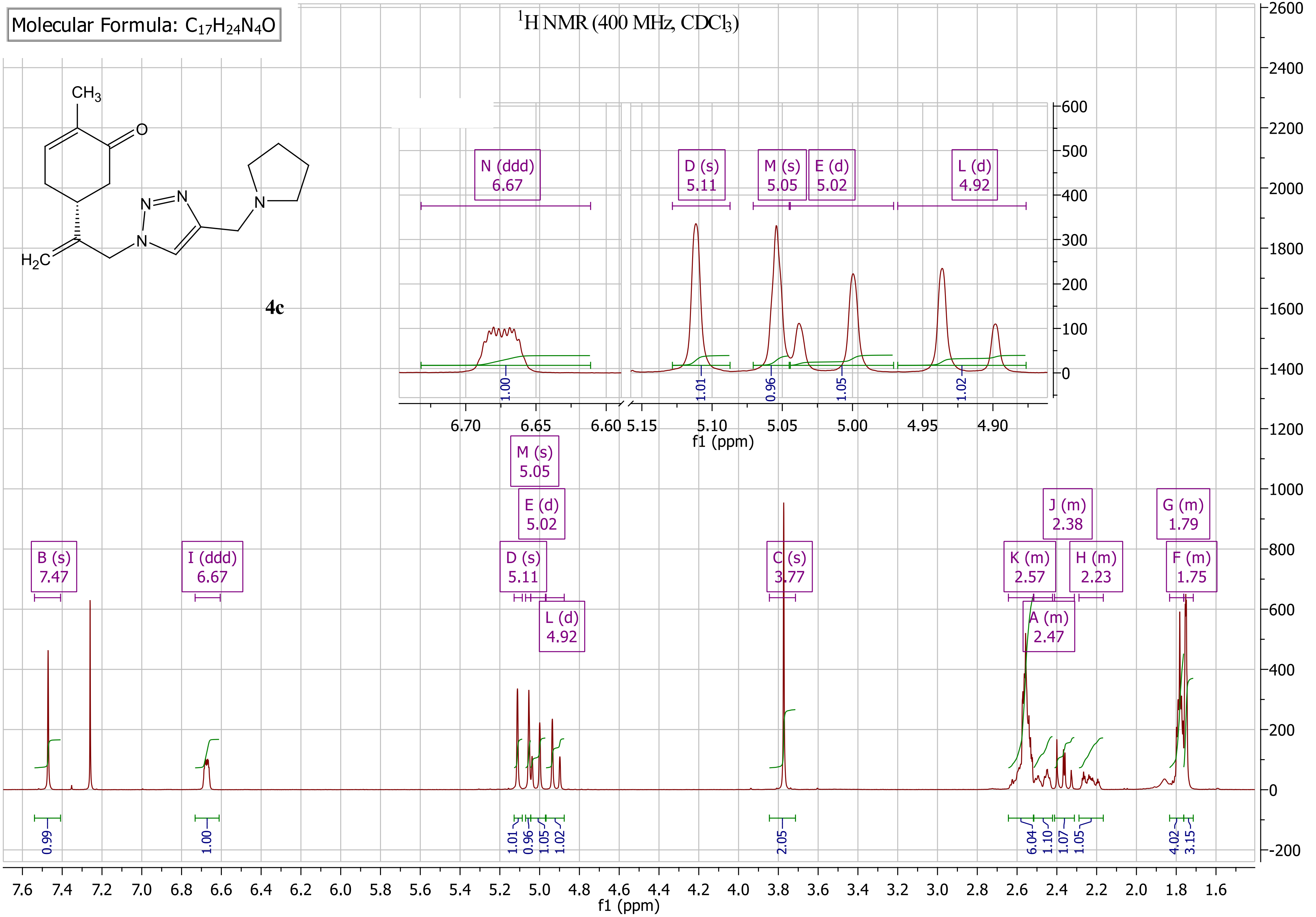

Molecular Formula: C17H24N4O
1H NMR (400 MHz, CDCl3)
CH3
O
N=N
N
N
H2C
4c
N (ddd) 6.67
D (s) 5.11
M (s) 5.05
E (d) 5.02
L (d) 4.92
1.00
1.01
0.96
1.05
1.02
6.70 6.65 6.60 5.15 5.10 5.05 5.00 4.95 4.90
f1 (ppm)
600 500 400 300 200 100 0
B (s) 7.47
I (ddd) 6.67
D (s) 5.11
M (s) 5.05
E (d) 5.02
L (d) 4.92
C (s) 3.77
K (m) 2.57
A (m) 2.47
J (m) 2.38
H (m) 2.23
G (m) 1.79
F (m) 1.75
0.99
1.00
1.01
0.96
1.05
1.02
2.05
6.04
1.10
1.07
1.05
4.02
3.15
7.6 7.4 7.2 7.0 6.8 6.6 6.4 6.2 6.0 5.8 5.6 5.4 5.2 5.0 4.8 4.6 4.4 4.2 4.0 3.8 3.6 3.4 3.2 3.0 2.8 2.6 2.4 2.2 2.0 1.8 1.6
f1 (ppm)
2600 2400 2200 2000 1800 1600 1400 1200 1000 800 600 400 200 0 -200

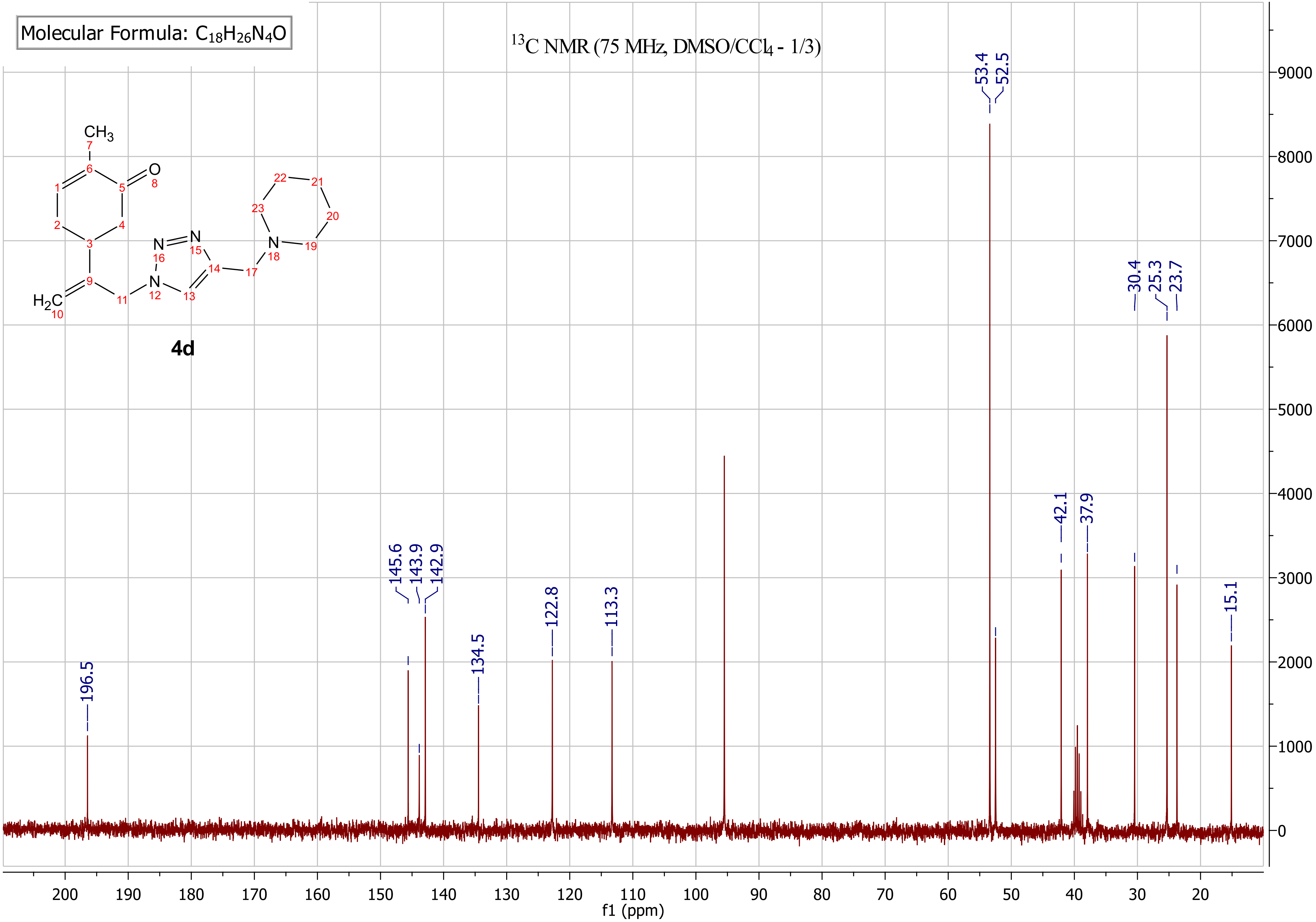

Molecular Formula: C18H26N4O
13C NMR (75 MHz, DMSO/CCl4 - 1/3)
4d
196.5
145.6
143.9
142.9
134.5
122.8
113.3
53.4
52.5
42.1
37.9
30.4
25.3
23.7
15.1
f1 (ppm)

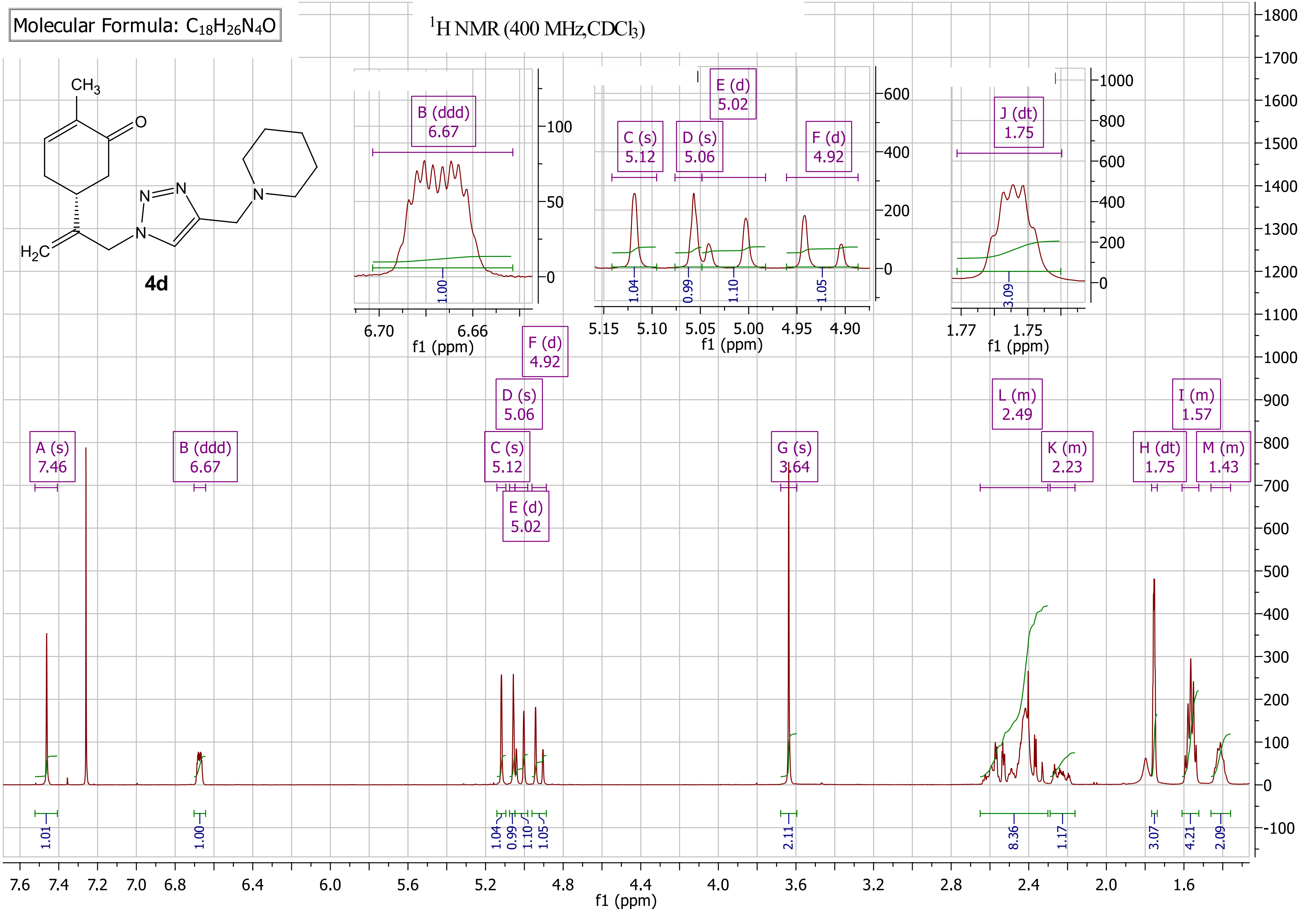

Molecular Formula: C18H26N4O
1H NMR (400 MHz,CDCl3)
CH3
O
N
N
N
N
H2C
4d
A (s) 7.46
B (ddd) 6.67
C (s) 5.12
D (s) 5.06
E (d) 5.02
F (d) 4.92
G (s) 3.64
H (dt) 1.75
I (m) 1.57
J (dt) 1.75
K (m) 2.23
L (m) 2.49
M (m) 1.43
1.01
1.00
1.04
0.99
1.10
1.05
2.11
8.36
1.17
3.07
4.21
2.09
3.09
f1 (ppm)

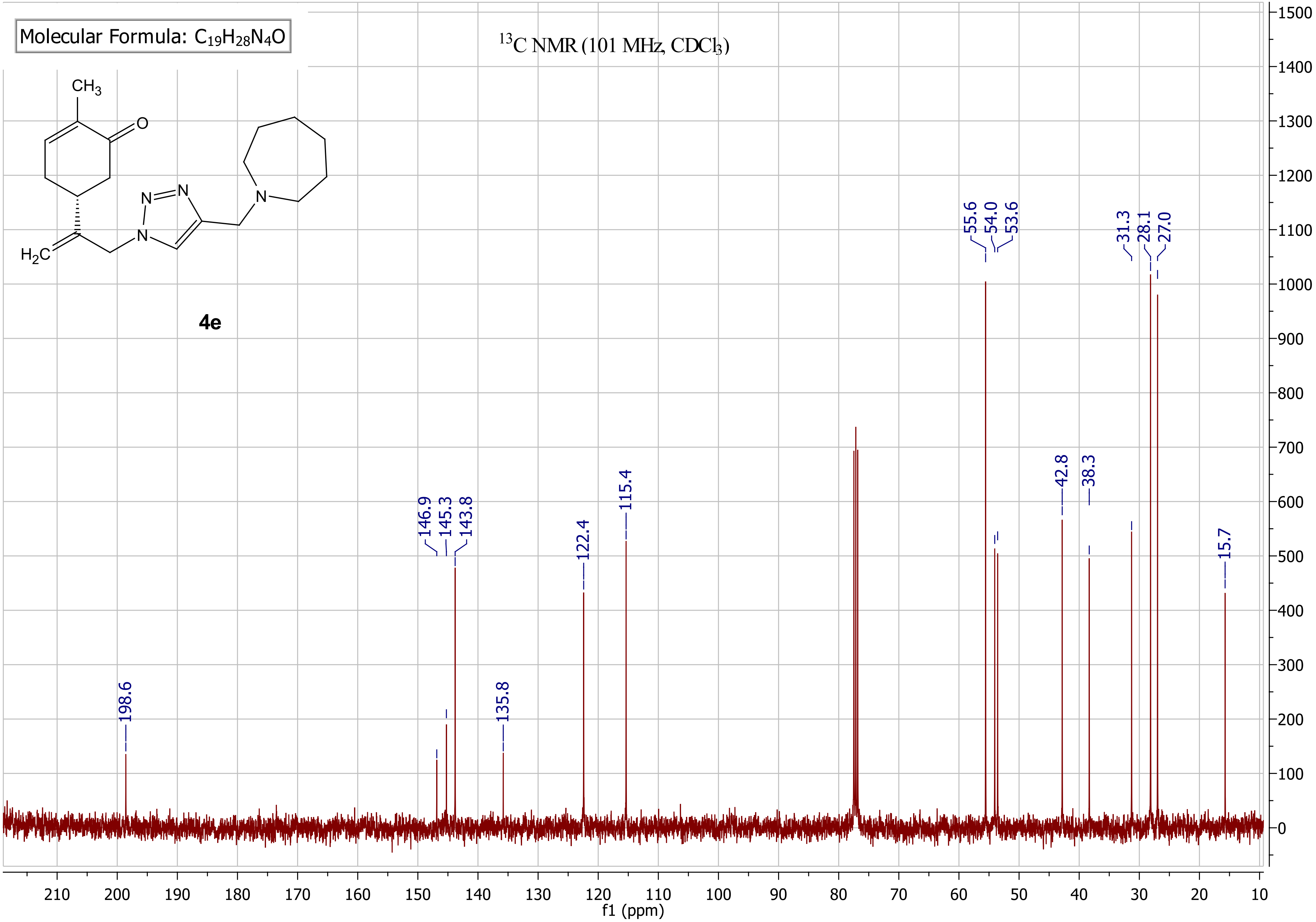
Molecular Formula: C19H28N4O
13C NMR (101 MHz, CDCl3)
4e
198.6
146.9
145.3
143.8
135.8
122.4
115.4
55.6
54.0
53.6
42.8
38.3
31.3
28.1
27.0
15.7
f1 (ppm)

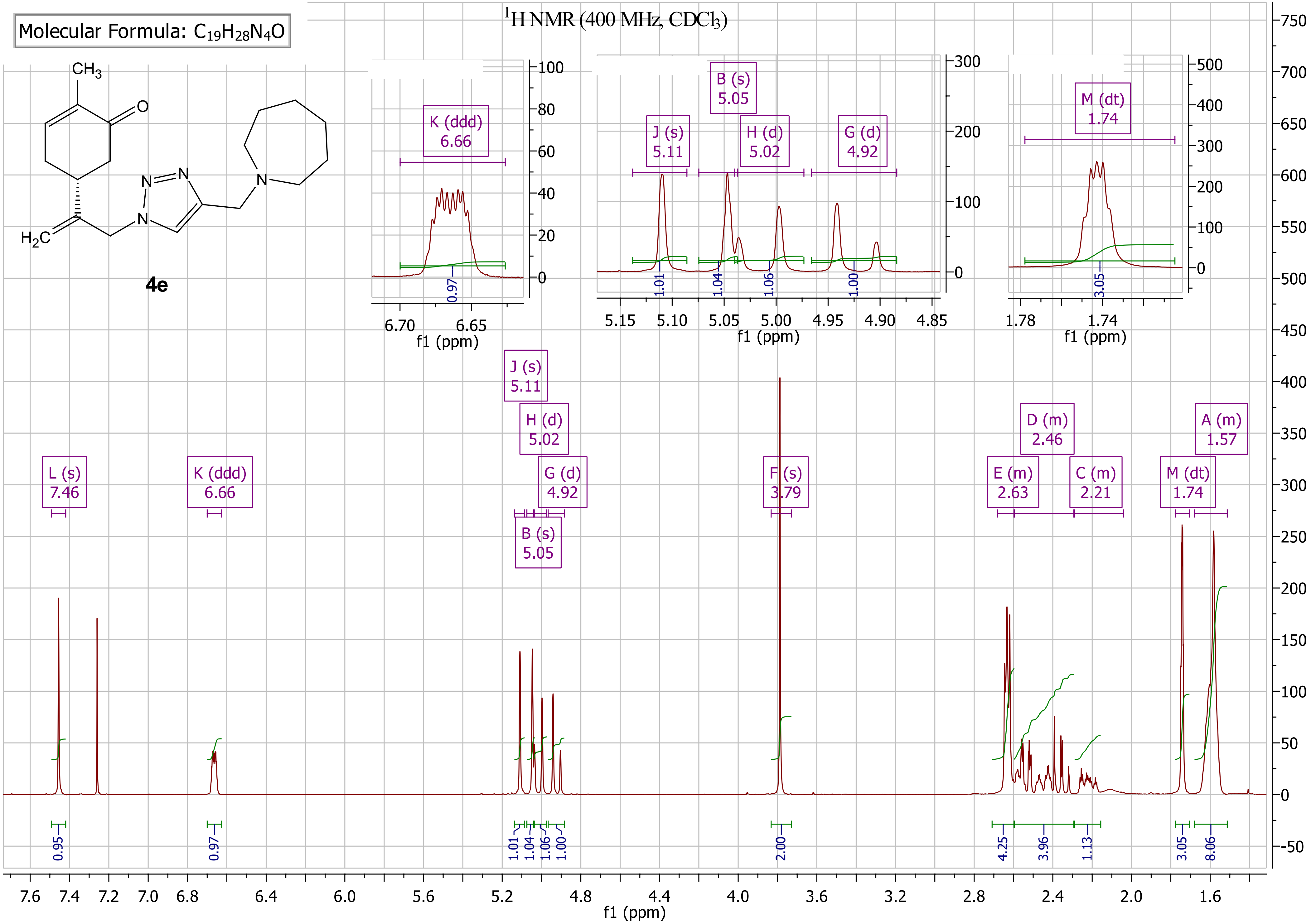

Molecular Formula: C19H28N4O
1H NMR (400 MHz, CDCl3)
4e
L (s) 7.46
K (ddd) 6.66
J (s) 5.11
B (s) 5.05
H (d) 5.02
G (d) 4.92
F (s) 3.79
E (m) 2.63
D (m) 2.46
C (m) 2.21
M (dt) 1.74
A (m) 1.57
0.95
0.97
1.01
1.04
1.06
1.00
2.00
4.25
3.96
1.13
3.05
8.06
f1 (ppm)

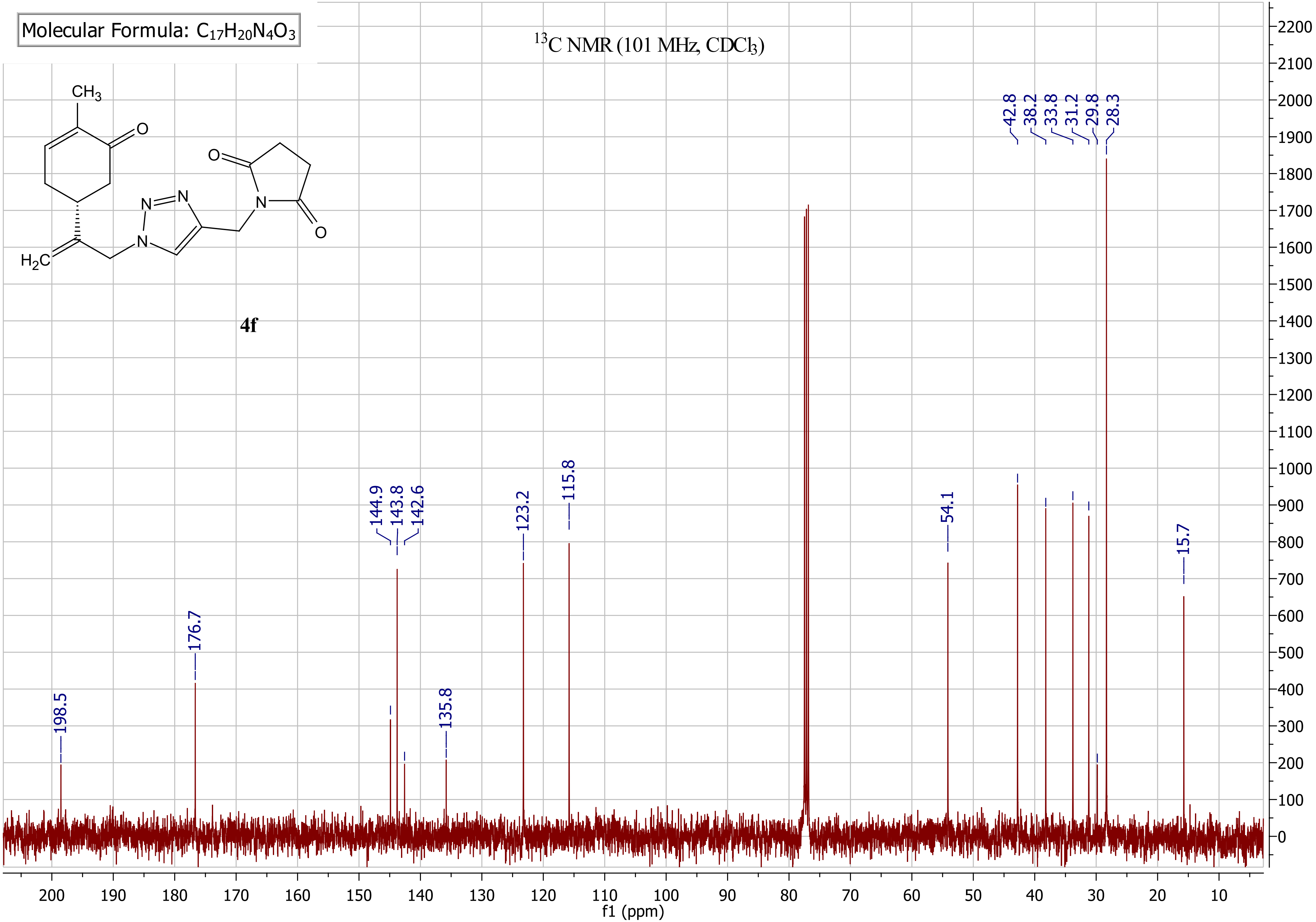

Molecular Formula: C17H20N4O3
13C NMR (101 MHz, CDCl3)
CH3
O
O
N
N
N
N
O
H2C
4f
198.5
176.7
144.9
143.8
142.6
135.8
123.2
115.8
54.1
42.8
38.2
33.8
31.2
29.8
28.3
15.7
f1 (ppm)

Molecular Formula: $C_{17}H_{20}N_4O_3$

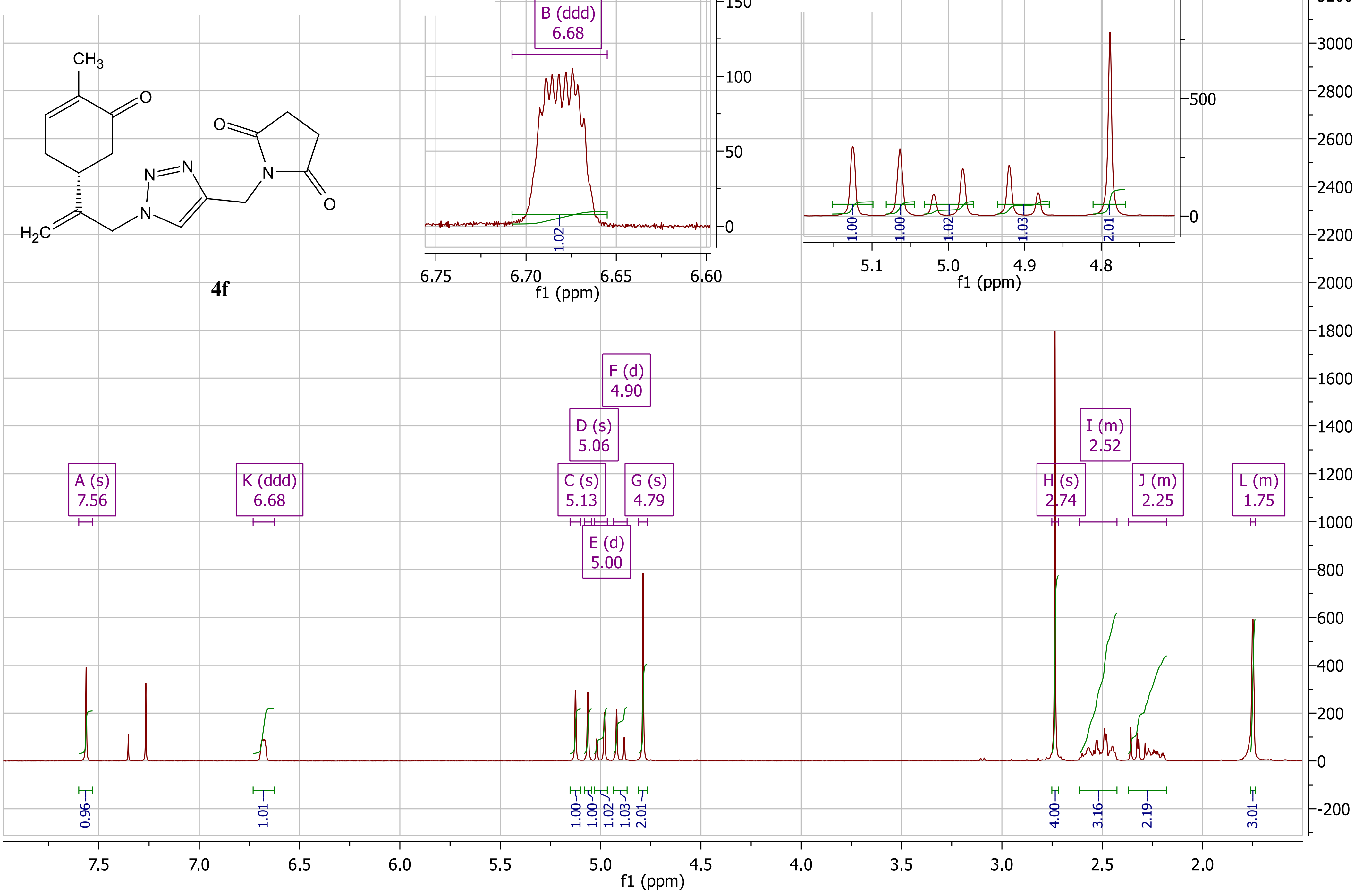

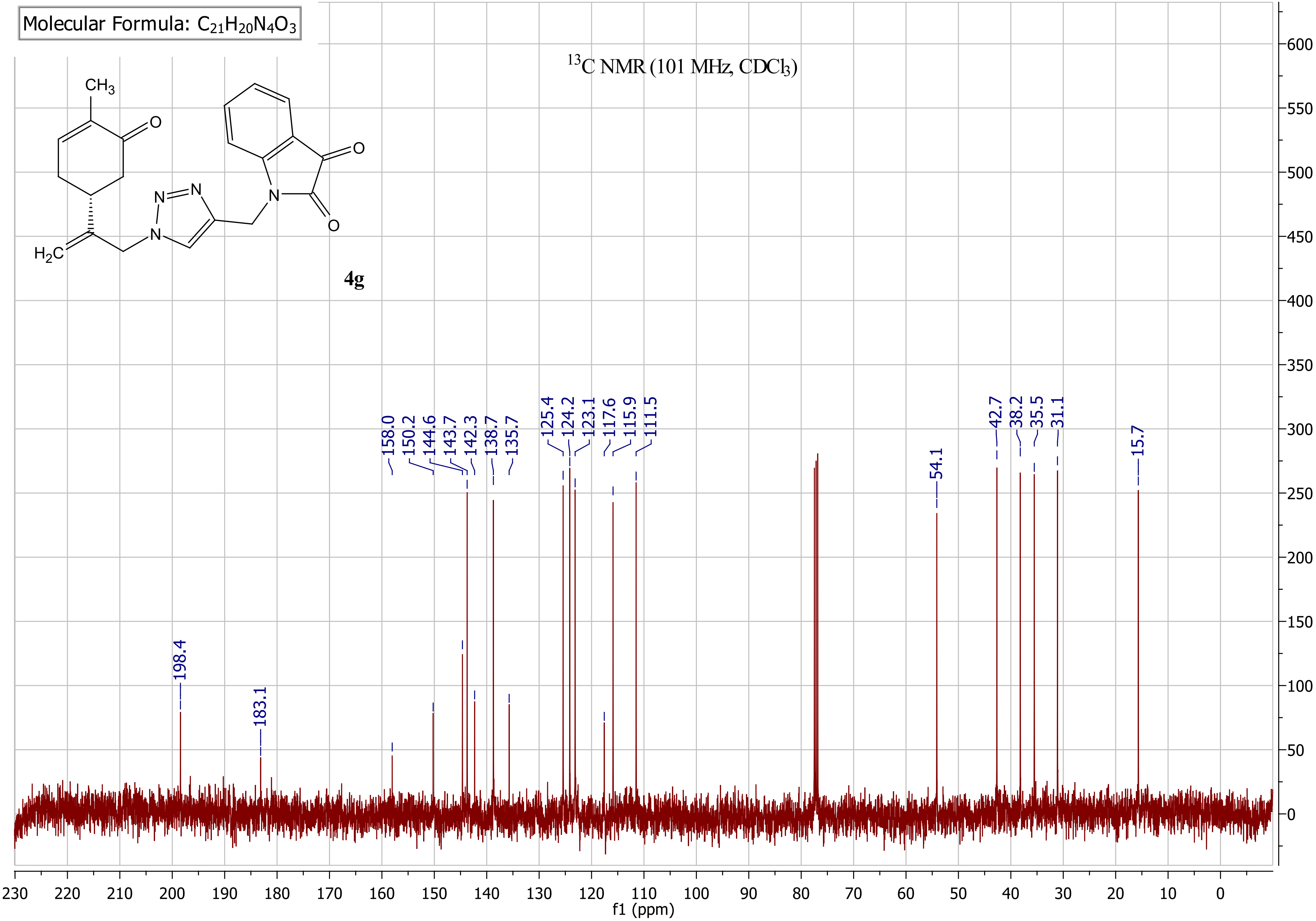

Molecular Formula: C21H20N4O3
13C NMR (101 MHz, CDCl3)
4g
198.4
183.1
158.0
150.2
144.6
143.7
142.3
138.7
135.7
125.4
124.2
123.1
117.6
115.9
111.5
54.1
42.7
38.2
35.5
31.1
15.7
f1 (ppm)

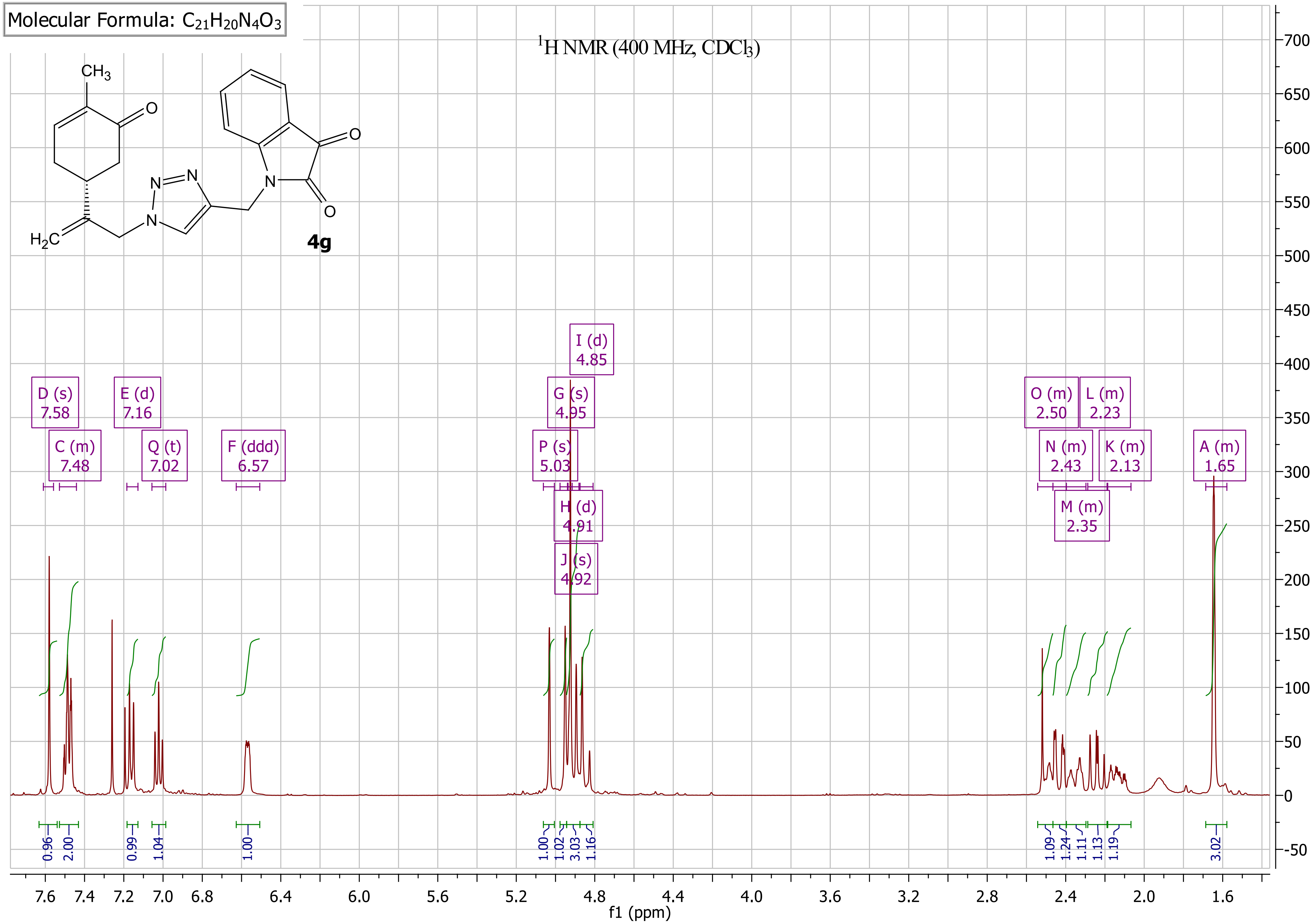

Molecular Formula: $C_{21}H_{20}N_4O_3$
^{1}H NMR (400 MHz, $CDCl_3$)
4g
D (s) 7.58
C (m) 7.48
E (d) 7.16
Q (t) 7.02
F (ddd) 6.57
P (s) 5.03
G (s) 4.95
I (d) 4.85
H (d) 4.91
J (s) 4.92
O (m) 2.50
L (m) 2.23
N (m) 2.43
K (m) 2.13
M (m) 2.35
A (m) 1.65
0.96 2.00 0.99 1.04 1.00 1.00 1.02 3.03 1.16 1.09 1.24 1.11 1.13 1.19 3.02
f1 (ppm)

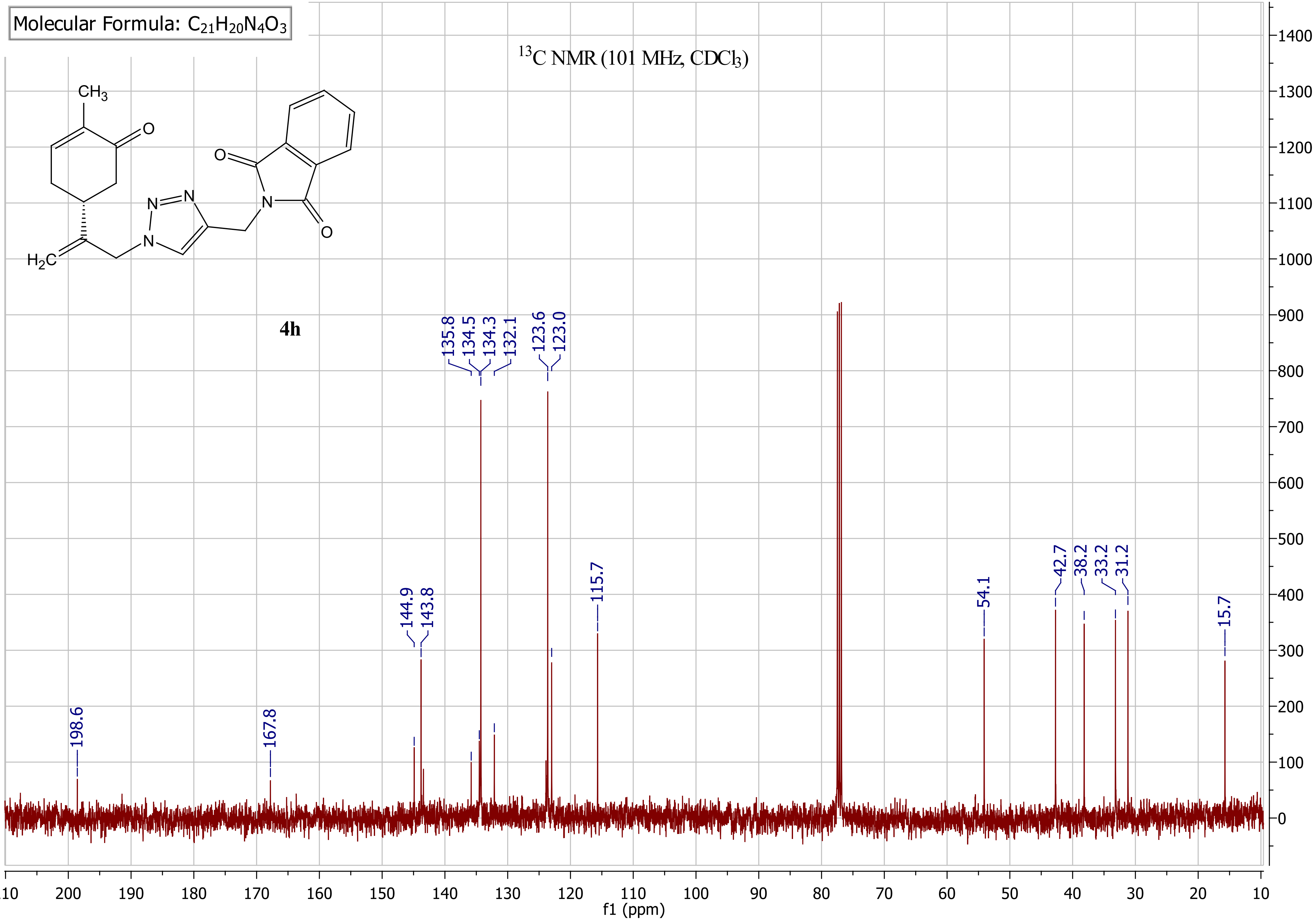

Molecular Formula: $C_{21}H_{20}N_4O_3$
^{13}C NMR (101 MHz, $CDCl_3$)
4h
198.6
167.8
144.9
143.8
135.8
134.5
134.3
132.1
123.6
123.0
115.7
54.1
42.7
38.2
33.2
31.2
15.7
f1 (ppm)

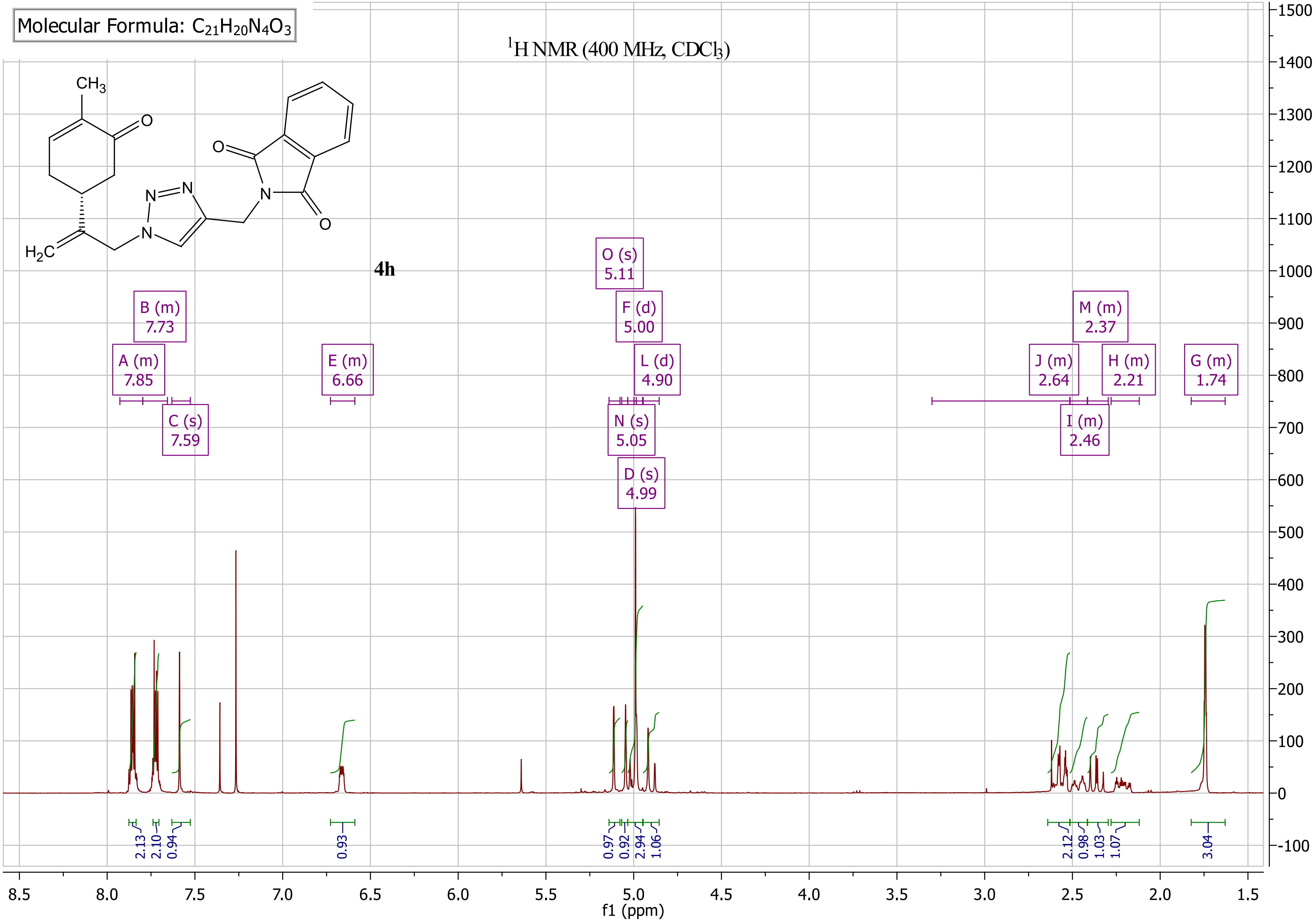
Molecular Formula: C21H20N4O3
1H NMR (400 MHz, CDCl3)
4h
O (s) 5.11
F (d) 5.00
B (m) 7.73
A (m) 7.85
E (m) 6.66
L (d) 4.90
C (s) 7.59
N (s) 5.05
D (s) 4.99
M (m) 2.37
J (m) 2.64
H (m) 2.21
G (m) 1.74
I (m) 2.46
2.13
2.10
0.94
0.93
0.97
0.92
2.94
1.06
2.12
0.98
1.03
1.07
3.04
f1 (ppm)

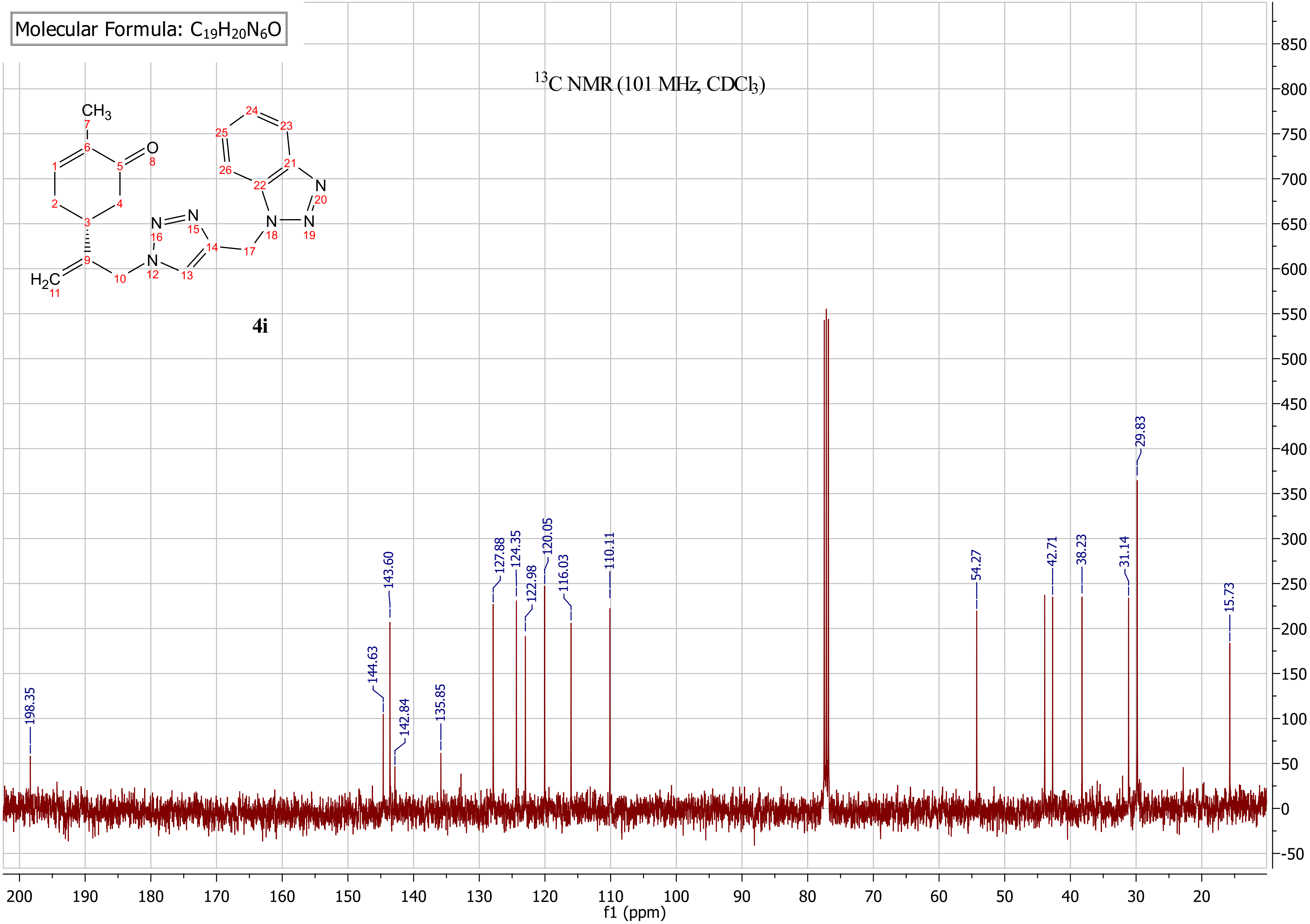

Molecular Formula: C19H20N6O
13C NMR (101 MHz, CDCl3)
4i
198.35
144.63
143.60
142.84
135.85
127.88
124.35
122.98
120.05
116.03
110.11
54.27
42.71
38.23
31.14
29.83
15.73
f1 (ppm)

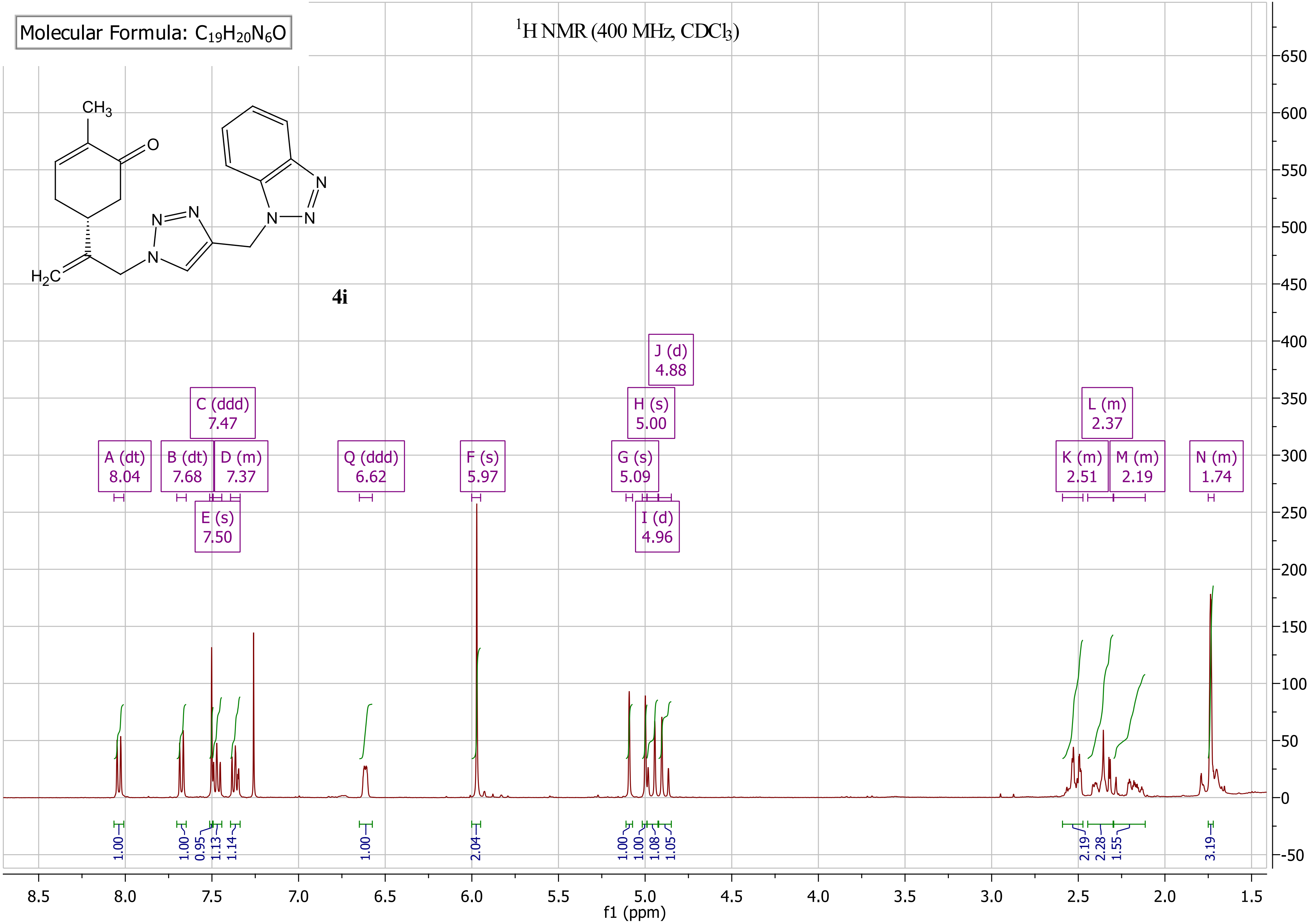

Molecular Formula: C19H20N6O
1H NMR (400 MHz, CDCl3)
CH3
O
N
N
N
N
N
N
H2C
4i
A (dt) 8.04
B (dt) 7.68
C (ddd) 7.47
D (m) 7.37
E (s) 7.50
Q (ddd) 6.62
F (s) 5.97
G (s) 5.09
H (s) 5.00
J (d) 4.88
I (d) 4.96
K (m) 2.51
L (m) 2.37
M (m) 2.19
N (m) 1.74
1.00
1.00
0.95
1.13
1.14
1.00
2.04
1.00
1.00
1.08
1.05
2.19
2.28
1.55
3.19
f1 (ppm)
